# *Pseudomonas putida* AlkA and AlkB Proteins Comprise Different Defense Systems for the Repair of Alkylation Damage to DNA – *In Vivo*, *In Vitro*, and *In Silico* Studies

**DOI:** 10.1371/journal.pone.0076198

**Published:** 2013-10-02

**Authors:** Damian Mielecki, Signe Saumaa, Michał Wrzesiński, Agnieszka M. Maciejewska, Karolina Żuchniewicz, Anna Sikora, Jan Piwowarski, Jadwiga Nieminuszczy, Maia Kivisaar, Elżbieta Grzesiuk

**Affiliations:** 1 Department of Molecular Biology, Institute of Biochemistry and Biophysics, Polish Academy of Sciences, Warsaw, Poland; 2 Department of Genetics, Institute of Molecular and Cell Biology, University of Tartu, Tartu, Estonia; University of Massachusetts Medical School, United States of America

## Abstract

Alkylating agents introduce cytotoxic and/or mutagenic lesions to DNA bases leading to induction of adaptive (Ada) response, a mechanism protecting cells against deleterious effects of environmental chemicals. In *Escherichia coli*, the Ada response involves expression of four genes: *ada*, *alkA*, *alkB*, and *aidB*. In *Pseudomonas putida*, the organization of Ada regulon is different, raising questions regarding regulation of Ada gene expression. The aim of the presented studies was to analyze the role of AlkA glycosylase and AlkB dioxygenase in protecting *P. putida* cells against damage to DNA caused by alkylating agents. The results of bioinformatic analysis, of survival and mutagenesis of methyl methanesulfonate (MMS) or *N*-methyl-N’-nitro-*N*-nitrosoguanidine (MNNG) treated *P. putida* mutants in *ada*, *alkA* and *alkB* genes as well as assay of promoter activity revealed diverse roles of Ada, AlkA and AlkB proteins in protecting cellular DNA against alkylating agents. We found AlkA protein crucial to abolish the cytotoxic but not the mutagenic effects of alkylans since: (i) the mutation in the *alkA* gene was the most deleterious for MMS/MNNG treated *P. putida* cells, (ii) the activity of the *alkA* promoter was Ada-dependent and the highest among the tested genes. *P. putida* AlkB (PpAlkB), characterized by optimal conditions for *in vitro* repair of specific substrates, complementation assay, and M13/MS2 survival test, allowed to establish conservation of enzymatic function of *P. putida* and *E. coli* AlkB protein. We found that the organization of *P. putida* Ada regulon differs from that of *E. coli*. AlkA protein induced within the Ada response is crucial for protecting *P. putida* against cytotoxicity, whereas Ada prevents the mutagenic action of alkylating agents. In contrast to *E. coli* AlkB (EcAlkB), PpAlkB remains beyond the Ada regulon and is expressed constitutively. It probably creates a backup system that protects *P. putida* strains defective in other DNA repair systems against alkylating agents of exo- and endogenous origin.

## Introduction

Alkylating agents of endogenous (by-products of cellular metabolism) and exogenous (environmental chemicals) origin introduce a variety of damages to DNA. The major products of alkylation include *N* ^7^-methylguanine (7meG), *N* ^3^-methyladenine (3meA) and *O* ^6^-methylguanine (*O* ^6^meG) with smaller amounts of *N* ^1^-methyladenine (1meA), *N* ^3^-methylcytosine (3meC), *O* ^4^-methylthymine (*O* ^4^meT), and methylphosphotriesters (MPTs). S_N_2 type alkylating agents, such as methyl methanesulfonate (MMS), predominantly produce *N*-methylation, whereas the S_N_1 type, such as *N*-methyl-N’-nitro-*N*-nitrosoguanidine (MNNG), create both *N*- and *O*-methylation [1]. Methylated bases can be toxic to the cell by blocking replication (e.g. 3meA, 1meA, 3meC, 3meG), can induce mutations (e.g. *O* ^6^meG, 3meC, 3meA, 1meA) or are innocuous (e.g. 7meG). Additionally, the purines methylated at N^3^ or N^7^ position can be easily hydrolyzed creating abasic (AP) sites that, if not repaired, are toxic or mutagenic to the cell [2-4].

Organisms are well equipped with mechanisms protecting cells against harmful effects of lesions introduced by alkylating agents. In *E. coli*, these defense systems involve the *tag* and *ogt* genes, expressed constitutively, and *ada*, *alkA*, *alkB*, and *aidB*, expressed after induction of the adaptive response (Ada response) [5-7]. A crucial role in this process is played by *ada*-encoded Ada methyltransferase, functioning as a transcriptional activator inducing expression of the *ada*-*alkB* operon and the *alkA* and *aidB* genes. Ada protein is composed of two major domains: the 19 kDa C-terminal AdaA domain (C-Ada19), directly demethylating *O* ^6^meG and *O* ^4^meT and transferring the methyl groups onto its own cysteine 321 (Cys-321) [8], and the 20 kDa N-terminal AdaB domain, demethylating S_p_-diastereoisomers of MPTs in DNA by transferring the methyl groups onto its cysteine 38 (Cys-38). Self-methylation at Cys-38 results in transcriptional activation of Ada protein and in an increase in specific DNA binding affinity to genes containing the sequence of *ada* operator in their promoters. High concentration of unmethylated Ada protein (>200 molecules per cell) inhibits transcriptional activation [9], whereas MPTs act as molecular sensors for changing the levels of DNA alkylation in bacteria [5]. Induced within the Ada response AlkA and expressed constitutively Tag proteins are, respectively, 3meA DNA glycosylase II and I. By removing 3meA, these glycosylases create AP sites in DNA, subsequently repaired by excision of the damaged fragment, re-synthesis and ligation of the DNA breaks. The specificity of AlkA is much broader than that of Tag. Besides 3meA, AlkA also removes 7-methyladenine (7meA), 3-methylguanine (3meG), 7meG, products of nitrosation, e.g. xanthine and oxanine [10], and some other types of alkylated bases [11]. It has also been found that AlkA (but not Tag) can remove normal bases (mainly G) from DNA [12]. Recently, it has been proven that AlkAs from *Archaeoglobus fulgidus* [13] and *Deinococcus radiodurans* [14] additionally show activity towards the main substrates of AlkB dioxygenase, 3meC and 1meA.

AlkB dioxygenase directly regenerates unmodified bases from the N^1^ position of adenine and N^3^ of cytosine [15,16]. Using non-heme Fe(II) and co-substrates, 2-oxoglutarate (2OG) and oxygen (O_2_), it initiates oxidative demethylation of DNA/RNA bases [17]. *E. coli* AlkB (EcAlkB) in the presence of O_2_ converts 2OG to succinate and CO_2_. The initial hydroxylation of the methyl group results in cleavage of the C-N bond restoring unmodified A and C bases both, in DNA and RNA. N^1^ of A and N^3^ of C are much more susceptible to methylation in single-stranded (ss) than in double-stranded (ds) DNA and, consequently, AlkB repairs lesions in ssDNA more efficiently than in dsDNA [18]. Similarly, AlkB oxidizes ethyl, propyl, hydroxyethyl and hydroxypropyl modifications. Recently, it has been found that it repairs also exocyclic adducts that may arise as a consequence of endocellular oxidative stress or environmental pollution – ethano- (3,*N* ^4^-α-hydroxyethanocytosine, HEC), etheno- (1,*N* ^6^-ethenoadenine, εA and 3,*N* ^4^-ethenocytosine, εC) and hydroxypropano- (3,*N* ^4^-α-hydroxypropanocytosine, HPC) groups added to DNA bases [19-22]. 3-methylthymine (3meT) and 1-methylguanine (1meG) are also repaired by AlkB but much less efficiently than 1meA or 3meC [20,21].

Our bioinformatic analysis has shown the presence of *E. coli* AlkB homologs in almost all organisms [23]. Higher eukaryotes possess several dioxygenases (e.g. nine in human [24,25], 13 in *Arabidopsis thaliana* [23]) of different cell localization, biological functions and substrate specificity.

The role of AidB, the fourth member of the Ada response, remains unclear. The protein preferentially binds AT-rich transcription enhancer sequences (UP elements), found upstream of many highly expressed genes, and seems to protect DNA from damage by alkylating agents [26].

The Ada response is conserved among many bacterial species although domains of Ada and AlkA proteins occur in diverse combinations in different prokaryotes. In the present study we investigated the organization of Ada response in *Pseudomonas putida*. The genus *Pseudomonas* constitutes a large diverse group of mostly saprophytic bacteria inhabiting soil, water, plants and animals, and playing an important role in community of soil microorganisms. These bacteria are able to metabolize toxic organic materials and are tolerant to antibiotics, organic solvents and heavy metals [27-29].

Here, we report *in silico*, *in vitro* and *in vivo* studies on the role of *P. putida* AlkA and AlkB proteins in protecting cellular DNA against alkylation lesions. In *P. putida* cells AlkA peremptorily plays a crucial role in repairing MMS and MNNG induced lesions in DNA, as evidenced by: (i) lack of AlkA glycosylase resulting in strong cytotoxic effect in MMS/MNNG-treated *P. putida alkA* mutant; (ii) wider than for *E. coli* AlkA substrate specificity probably including typical AlkB substrates, 1meA and 3meC (iii) the highest among *P. putida* Ada genes activity of *alkA* promoter in the presence of Ada protein. We have found *P. putida* and *E. coli* AlkB proteins to be similar in substrate specificity but differently regulated. The results of promoter activity allow to predict constitutive expression of Pp*alkB*, suggesting its supportive role in protecting cells against exo- and endogenous alkylating agents.

## Results

### The genomes of *P. putida* species code more putative proteins dealing with alkyl-DNA damages than *E. coli*

**Figure 1:**
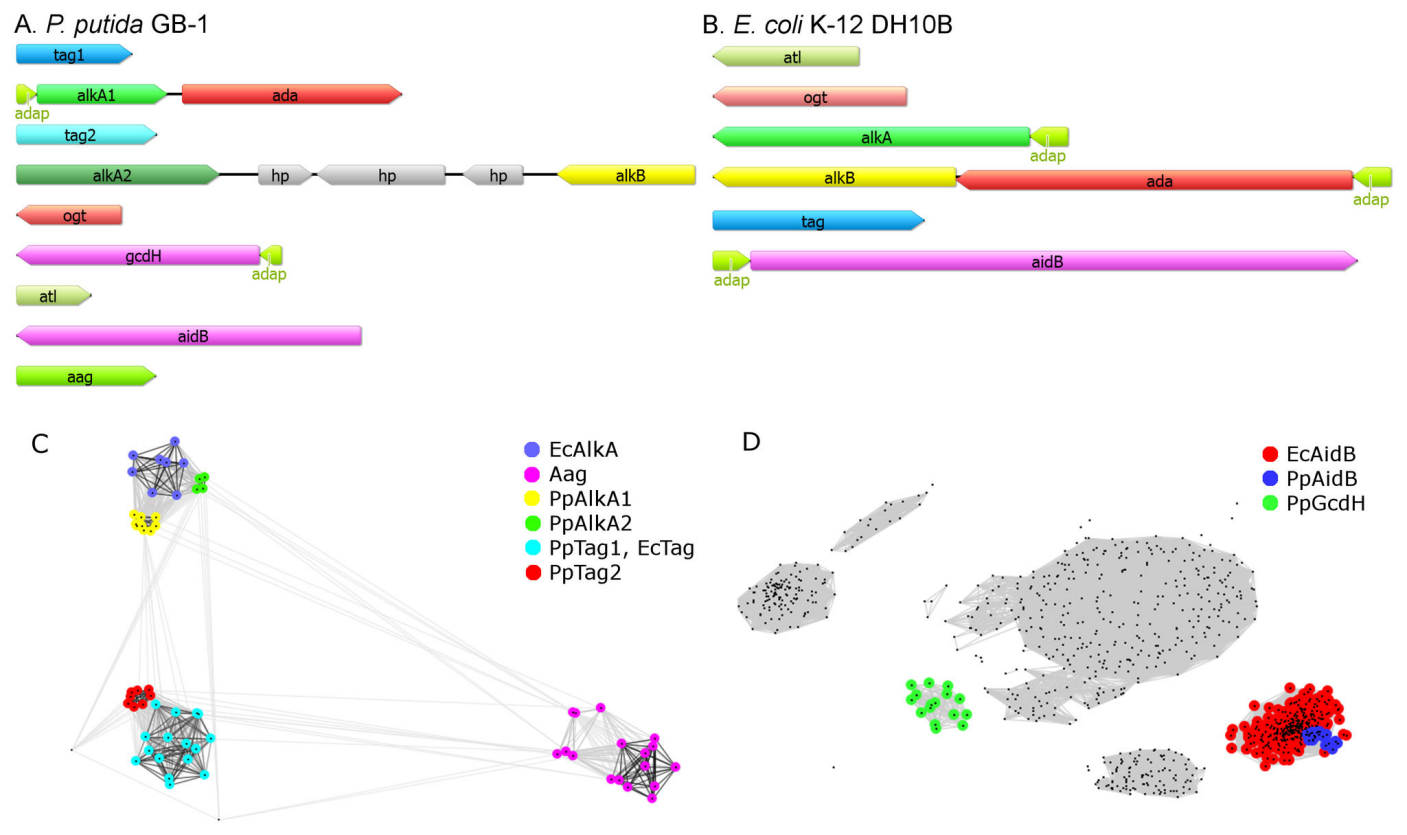
The genes encoding proteins involved in the repair of alkylation damage to DNA in (A) *P. putida* (*tag1* – locus tag: PputGB1_0078, *alkA* – PputGB1_0739, *ada* – PputGB1_0740, *tag2* – PputGB1_1244, *alkA2* – PputGB1_2545, *alkB* – PputGB1_2549, *ogt* – PputGB1_2805, *gcdH* – PputGB1_3505, *atl* – PputGB1_4493, *aidB* – PputGB1_4834, *aag* – PputGB1_4865, *ada*p – promoter region containing putative Ada box, hp – hypothetical protein) and (B) *E. coli* species (*atl* – ECDH10B_0410, *ogt* – ECDH10B_1455, *alkA* – ECDH10B_2218, *alkB* – ECDH10B_2369, *ada* – ECDH10B_2370, *tag* – ECDH10B_3728, *aidB* – ECDH10B_4382). The CLANS protein clustering of *P. putida* and *E. coli* AlkA, Tag, and Aag sequences (C) indicates that PpAlkA2s are 3meA glycosylases most closely related to EcAlkA proteins. The CLANS clustering of *P. putida* and *E. coli* AidB protein sequences (family cd01154, NCBI) (D) clearly shows that the *P. putida* acyl-CoA dehydrogenases represented by *P. putida* KT2440 locus PP_4780 are most closely related to *E. coli* AidB proteins but the subfamily of PpGcdH, comprising other acyl-CoA dehydrogenases represented by *P. putida* KT2440 locus PP_0158, preserve perfect Ada boxes in their promoter regions (see Figure 3).

In *E. coli*, the adaptive response results in increased expression of four genes, *ada*, *alkB*, *alkA* and *aidB* [5,6]. In order to establish whether a similar response occurs in pseudomonads, we searched for the presence of *E. coli* Ada regulon orthologs in *P. putida* genome and examined the ability of alkylating agents to induce transcription of these genes. The orthologs for *E. coli* Ada regulon genes are present in the genome of *P. putida* KT2440 (genome analysis, NCBI database). However, the organization of *alkA* (PP_0705), *ada* (PP_0706) and *alkB* (PP_3400) orthologs in *P. putida* differs from that of *E. coli*. In *E. coli*, the *ada* and *alkB* genes comprise one operon, separated by 160 kbp from *alkA*, whereas in *P. putida*, the *alkA* and *ada* genes are located together (and are most probably transcribed in the direction *alkA-ada*), and the *alkB* gene is located about 3 Mbp apart (Figure 1A and B).

**Figure 2:**
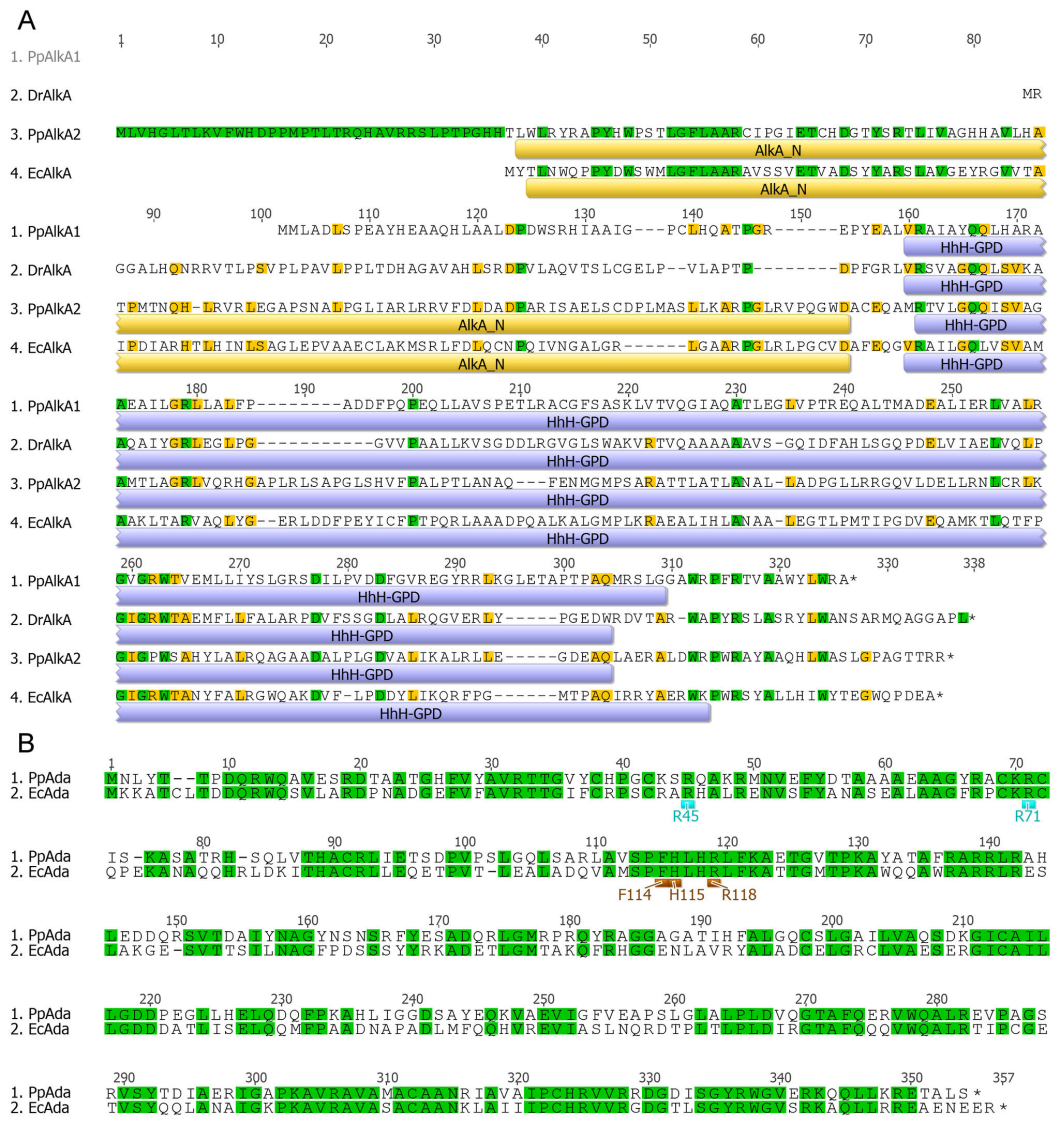
The multiple protein alignment of *E. coli* K-12 DH10B EcAlkA (locus tag: ECDH10B_2218), *P. putida* KT2440 PpAlkA (PP_0705), *P. putida* GB-1 PpAlkA2 (PputGB1_2545), and *D. radiodurans* DrAlkA (DR_2584) generated with ClustalW (A). Yellow and blue bars represent Pfam domains corresponding to the above sequence: AlkA_N – the N-terminal domain of 3meA glycosylase AlkA proteins; HhH-GPD – helix-hairpin-helix domain with Gly/Pro rich loop followed by a conserved aspartate residue. The multiple protein alignment of *P. putida* KT2440 PpAda (PP_0706) and *E. coli* K-12 DH10B EcAda (ECDH10B_2370) proteins (B) shows the perfect conservation of residues responsible for the specific interaction with the conserved nucleotide sequences of the *ada* regulon promoters: R45 and R71 (light blue) binding to the A box and F114, H115, and R118 (brown) binding to the B box.

The predicted amino acid sequences of *P. putida* KT2440 and *E. coli* K12 DH10B Ada proteins exhibited 54.6% of identity (Figure 2B) and those of AlkB 56% identity. PpAda sequence contains all of the amino acids homologous to the EcAda residues responsible for the sequence-specific DNA binding to the *E. coli ada* regulon promoters, R45 and R71, for the binding to the conserved A box (AAT), and F114, H115 and R118, for the specific interactions with the B box (GCAA) of these promoters [30]. This indicated that the mechanisms of transcriptional activation by Ada proteins are conserved between *E. coli* and *P. putida*.

PpAlkB protein contains all of the amino acid residues required for including this protein into the dioxygenase superfamily. Our latest clustering image allowed to place PpAlkB into the first subgroup, together with EcAlkB and other proteobacterial AlkBs [23].

Unlike Ada and AlkB proteins, EcAlkA and PpAlkA sequences are largely different. The predicted amino acid sequence identity between these proteins equals only 17.2% (Figure 2A). Furthermore, PpAlkA is shorter by 61 amino acid residues at its N-terminus. Consequently, it lacks the N-terminal α/β domain present in EcAlkA. Nevertheless, the PpAlkA protein still consists of the C-terminal glycosylase domain and the vital catalytic aspartate residue. Moreover, it resembles *Deinococcus radiodurans* AlkA (DrAlkA) with 33.5% identity [14], which could determine the different mechanism of action and/or substrate specificity of this protein in comparison to EcAlkA. Interestingly, several other analyzed *P. putida* genomes (e.g., *P. putida* strains GB-1, S16, HB326, and NBRC14164) contain additional *alkA*, named here *alkA2* (Figure 1A and B). The PpAlkA2 proteins are full-length putative 3meA glycosylases composed of two AlkA domains and are mostly similar to EcAlkA (Figure 1C) exhibiting about 34% of identity (Figure 2A).

**Figure 3:**
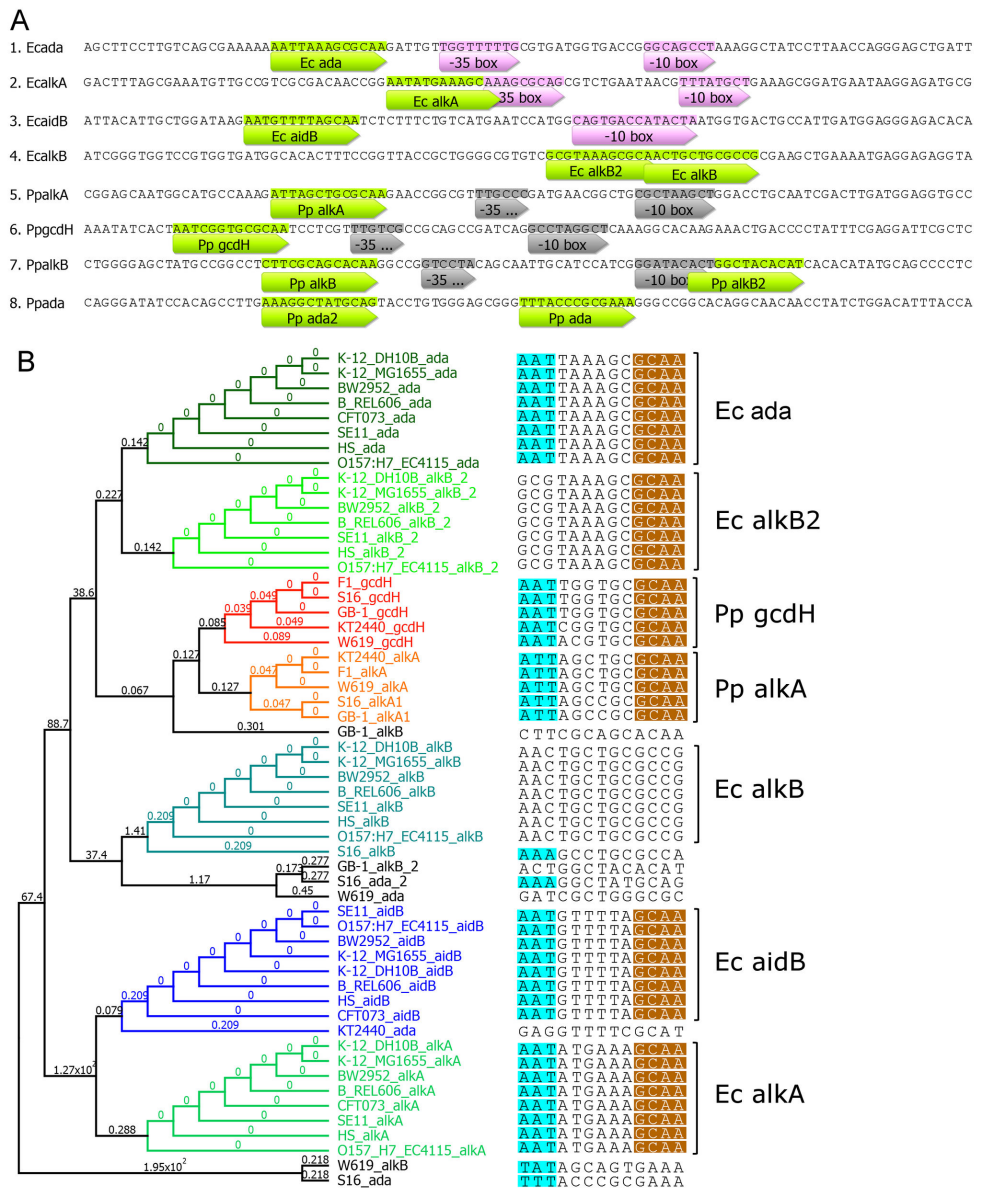
The consensus sequences responsible for Ada protein specific interaction (A) located in promoter sequences (up to -100 nucleotide residues relative to ATG start codons) of *E. coli ada*, *alkA*, and *aidB*. Additionally, the predicted consensuses in promoter regions of *E. coli* *alkB* and *P. putida* *alkA*, *gcdH*, *alkB*, and *ada* genes are shown (green bars). Also, the putative -35 and -10 boxes responsible for RNA polymerase interaction are marked in pink (retrieved from RegulonDB, http://regulondb.ccg.unam.mx) or grey (SoftBerry BPROM prediction tool, http://linux1.softberry.com). The phylogenetic tree (B) shows relative conservation between depicted consensus sequences based on UPGMA Geneious Tree Builder, A box – light blue, B box – brown, numbers indicate substitutions per site (for details see Materials and Methods).

The AidB family (cd00540) contains the mean number of 15 and 4 AidB proteins for *P. putida* and *E. coli* species, respectively. Since the acyl-CoA dehydrogenases are highly similar, it is difficult to precisely identify putative functional *P. putida* AidB gene. The protein clustering of *P. putida* and *E. coli* acyl-CoA dehydrogenases shows that the proteins represented by *P. putida* KT2440 PP_4780 locus belong to the subfamily of functional *E. coli* AidB proteins (Figure 1D), but, as shown below, the promoter regions of these genes do not contain putative Ada boxes. Instead, other proteins of *P. putida* acyl-CoA dehydrogenases subfamily represented by *P. putida* KT2440 PP_0158 locus (Figure 1D) are coded by genes with perfectly preserved Ada box sequences in their promoter regions (Figure 3B). Moreover, this locus was obtained as a result of genomic context search in MicrobesOnline database using *E. coli aidB* as a query. These proteins are annotated as glutaryl-CoA dehydrogenases and named here GcdH. Since the precise function of AidB is unknown, we focused our functional studies only on *alkA*, *ada*, and *alkB* orthologs.

In addition to the potential *ada* regulon genes, all of the analyzed *P. putida* genomes show the presence of the second *tag* gene, named here as *tag2*. The protein clustering of putative repair glycosylases coping with alkyl-DNA damages shows that the additional copies of 3meA glycosylases of *P. putida* species – Tag2 and AlkA2 are mostly related to EcTag and EcAlkA proteins, respectively (Figure 1C). The above list of *P. putida* genes can be widened by *aag* -3-alkylated purines and hypoxanthine DNA glycosylase (Figure 1A). Notably, both *P. putida* and *E. coli* genomes encode for Atl proteins, which have recently been identified as alkyltransferase-like proteins taking part in the repair of *O* ^6^-alkyl-guanines [31].

### *P. putida alkA* and *gcdH* Gene Promoters Contain Canonical Ada Boxes

Particular nucleotide sequences in *E. coli ada*, *alkA* and *aidB* promoter regions responsible for specific interaction with Ada transcriptional activator were identified at the structural level [30]. To identify putative Ada boxes in the *P. putida* Ada regulon genes, motif searching and database scanning were performed (MEME Server, see Materials and Methods). The most conserved consensus sequences in *P. putida* were identified in the *gcdH* and *alkA* promoter regions. Both sequences contain canonical A and B boxes, AAT/ATT and GCAA, respectively, located at a similar distance from the -35/-10 RNA polymerase consensus, as in *E. coli ada* promoter region (Figure 3A). As expected, *E. coli alkB* promoter does not contain a fully preserved Ada box although the FIMO search identified two fragments with quite high similarity to *E. coli* and *P. putida* Ada box motifs. The same concerns *P. putida* putative *alkB* and *ada* promoter regions. Thus, degraded Ada box consensus can be speculated in Pp*alkB* promoter since it consists of putative -35/-10 RNA polymerase interaction sites. Actually, the *P. putida* S16 and W619 A boxes contain the demanded A/T base pairs. On the other hand, the B boxes show single nucleotide polymorphism with the GCCA and GAAA sequences in above mentioned strains, respectively (Figure 3B). Additionally, the *alkA2* genes of *P. putida* GB-1 and S16 strains do not show the Ada box motifs below the *P*-value of 0.01. The promoter regions of all 15 putative *P. putida* KT2440 acyl-CoA dehydrogenases were also tested for the presence of Ada box motifs with negative results (data not shown) except for the above mentioned *gcdH*.

### *P. putida alkA* promoter activity is higher than that of *alkB* and ada promoters

**Figure 4:**
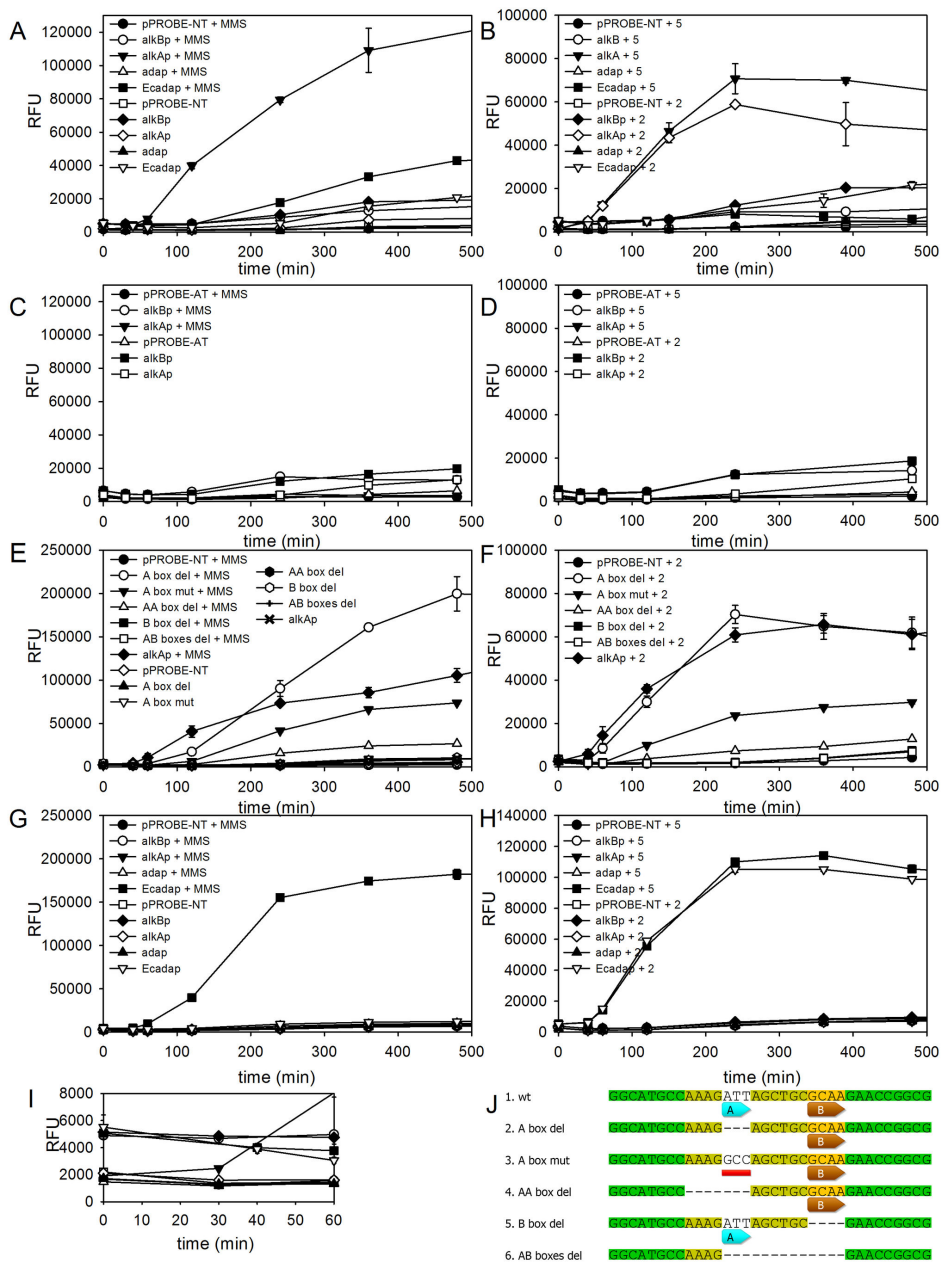
Promoter induction based on fluorescence intensities of GFP protein for the *P. putida* wt (A, B), *P. putida* ada*‾* (C, D) or *E. coli* AB1157 (G, H) strains treated with 20 mM MMS (A, C, G) or 2 or 5 µg/ml MNNG (B, D, H), also the graph for (A) showing the basal induction is included (I). The promoter activity for deletion or mutation derivatives of *P. putida* *alkA* promoter in the presence 20 mM MMS (E) or 2 µg/ml MNNG (F), with sequences changed as shown (J).

In order to examine whether in *P. putida* the transcription of the *alkB* gene and the putative *alkA-ada* operon is inducible by alkyl damage to DNA, the promoter activities of these genes were monitored with the use of a GFP reporter (expressed from the pPROBE-NT vector, see Materials and Methods). The fluorescence levels detected in *P. putida* plasmid-free cells or in cells carrying the pPROBE-NT empty vector expressed at the values of about 1-2 × 10^3^ RFU independently of MMS treatment (Figure 4). Similar basal level of fluorescence was detected in cells carrying the *gfp* transcriptional fusion with 100-bp DNA region derived upstream from the coding sequence of the *ada* gene, thereby implying that this DNA region does not contain any promoter for *ada* gene transcription. The transcriptional fusion with the DNA sequences located upstream of the *alkA* gene exhibited an increased level of fluorescence in the presence of MMS and MNNG. In MMS- or MNNG-treated cells carrying this construct, the level of fluorescence increased rapidly reaching 110 × 10^3^ or 70 × 10^3^ RFU, respectively, after 6 h of induction (Figure 4A and B). In non-treated control, the level of fluorescence did not exceed 8-10 × 10^3^ RFU staying almost uninduced in the course of the experiment. These results indicated the inducibility of a promoter located upstream of the *alkA* gene by DNA methylating agents. In order to find whether this activation is Ada-dependent, the expression of the Pp*alkA* promoter was measured in MMS/MNNG treated *P. putida* ada‾ strain. In ada‾ background, no significant induction of Pp*alkA* promoter was observed (Figure 4C and D).

On the other hand, at the start of the experiment, cells harboring the putative Pp*alkB* promoter fusion with the *gfp* reporter gene exhibited RFU level of about 5 × 10^3^, which is 2.6-fold higher than that in cells bearing other constructs. After 6 h, the level of RFU increased up to 20 × 10^3^ RFU regardless of MMS/MNNG treatment (Figure 4A, B, and I) reaching about 5.7-fold higher induction in comparison to cells with pPROBE-NT alone. Thus, in *P. putida*, the *alkB* promoter does not respond to DNA alkyl damage. *P. putida* cells carrying the pPROBE-NT vector lacking Pp*alkB* nucleotides -68 to -18 showed only a basal level of RFU (data not shown), probably because of the removal of the putative -35/-10 RNA polymerase boxes (Figure 3A). These results indicate that the DNA region lying between the indicated nucleotide positions comprises sequences of the Pp*alkB* promoter. Taken together, our results indicate that transcription regulation of Ada regulon in *P. putida* differs from the *E. coli* orthologs; only the expression of the *alkA*-*ada* operon is inducible by methylating agents and requires Ada for transcriptional activation, whereas the *alkB* gene is expressed constitutively.

To investigate the importance of putative A and B boxes in the Pp*alkA* promoter, particular deletion mutations were constructed (Figure 4J). Deletion of the B box as well as the entire fragment ranging from A to B box (13 nucleotides) in the Pp*alkA* promoter region completely diminished its inducible property regardless of MMS or MNNG treatment (Figure 4E and F). Quite opposite effect applies to the A box where the removal of ATT bases led to even stronger induction: after MMS treatment and 8 h of incubation it reached about 200 × 10^3^ RFU (Figure 4E). Nevertheless, the induction of transcription from this deletion mutant was delayed in comparison to the wild-type promoter. At the same time, the induction profile of this mutant promoter did not differ from that of the wild-type promoter in MNNG-treated cells (Figure 4F). Surprisingly, changing the ATT to GCC in the A box did not abolish the induction which reached about half of the value obtained for the wild-type *alkA* promoter (Figure 4E and F). Moreover, the complete lack of inducibility can be achieved by the deletion of additional AAAG bases upstream of the A box.

The *E. coli ada* promoter showed very strong induction in *E. coli* cells reaching about 180 and 110 × 10^3^ RFU after MMS or MNNG treatment, respectively. On the other hand, we have found that the *P. putida alkA* promoter cannot be induced in MMS- or MNNG-treated *E. coli* cells (Figure 4G and H). These results indicated that either the EcAda protein is not able to induce transcription from the *P. putida alkA* promoter or the activation of transcription from this promoter requires additional factors that are absent in *E. coli*. At the same time, the *E. coli ada* promoter was inducible in *P. putida* MMS-treated cells. However, the maximum level of induction of this promoter in *P. putida* was about 3-fold lower (40 × 10^3^ RFU) in comparison to that in *E. coli* cells (compare Figure 4A and G).

### AlkA protein allows *P. putida* survival in the presence of alkylating agents

**Figure 5:**
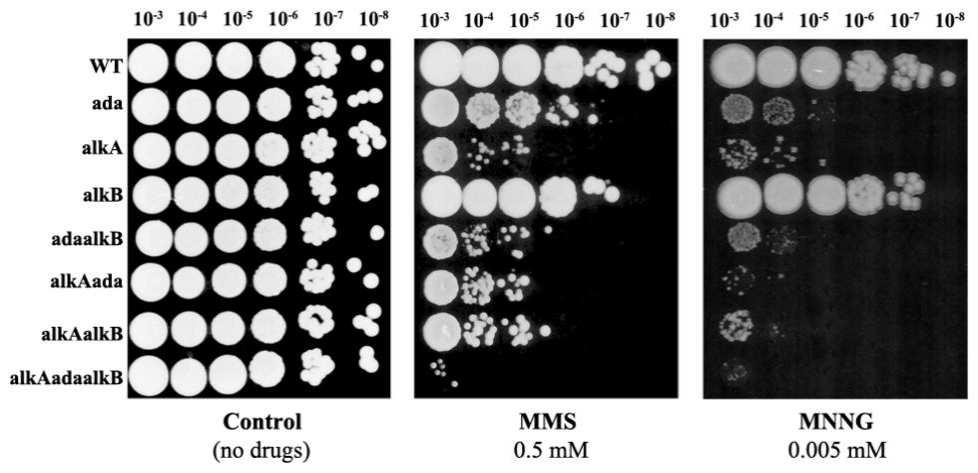
Sensitivity of *P. putida* AlkA-, AlkB-, and Ada-deficient mutants to alkylating chemicals MMS and MNNG. The serial dilution drop test of *P*. *putida* mutants: 1 – wild-type, 2 – alkB‾, 3 – alkA‾, 4 – ada‾, 5 – alkB‾ada‾, 6 – alkA‾ada‾, 7 – alkA‾alkB‾, 8 – alkA‾alkB‾ada‾ on the medium containing the indicated concentrations of MMS (A) or MNNG (B). The vertical arrow shows the direction of dilution: from 10^-1^ to 10^-8^.

In order to recognize the impact of AlkA, AlkB and Ada on protection of *P. putida* cells against alkylating agents, *P. putida* wild-type strain and its derivatives lacking *alkA*, *alkB* or ada genes separately (single mutants) or in combinations (double and triple mutants) were compared for survival after MMS or MNNG treatment. Since MMS methylates mainly *N* atoms and MNNG *N* and *O* atoms in DNA bases [1], we could expect differences in sensitivity of *P. putida* mutants to these alkylating agents. At first, we performed a drop test with serial dilutions (10^-3^ to 10^-8^) of bacterial cultures spotted on agar plates containing (or not, for the control) 0.5 mM MMS or 0.74 µg/ml MNNG. These concentrations of MMS and MNNG did not affect significantly the survival of the wild-type *P. putida* and its AlkB-deficient derivative (Figure 5). The absence of AlkA strongly affected survival of bacteria on MMS and MNNG plates; moreover, the *alkA* ^-^ bacteria were more sensitive to MNNG than to MMS. The Ada-deficient *P. putida* was only slightly more sensitive to MMS than the wild-type, but there was a dramatic reduction in the survival of bacteria on MNNG plates. Requirement for AlkB became evident only in *P. putida* alkA‾ and ada‾alkA‾ mutants. This meant that the survival of the ada‾alkB‾ double mutant was reduced in comparison to the ada‾ single mutant, and the survival of the ada‾alkA‾alkB‾ triple mutant was reduced in comparison to the ada‾alkA‾ double mutant.

**Figure 6:**
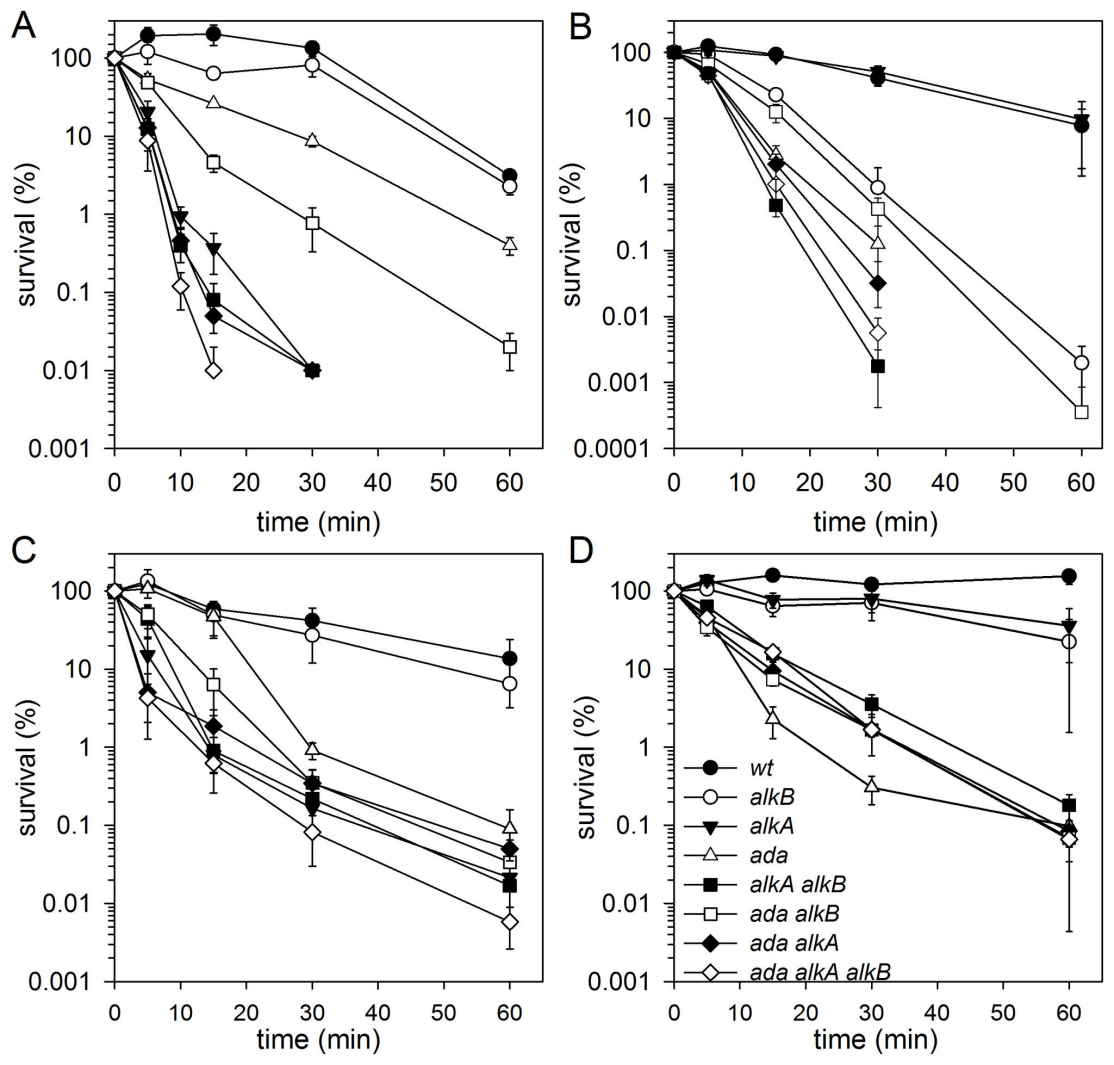
Survival of *P. putida* (A, C) and *E. coli* (B, D) mutants defective in *alkA*, *alkB*, and *ada* genes treated with 20 mM MMS (A, B) or 5 µg/ml MNNG (C, D) for the indicated time.

To monitor more precisely the dynamics of the survival of *P. putida* wild-type strain and its Ada-, AlkA-, and AlkB-deficient derivatives, the bacteria in liquid cultures were treated with MMS or MNNG and samples were taken at different time points to monitor cell survival. In this assay, *P. putida* alkB‾ mutant survived MMS treatment similarly as the wild-type strain, and mutation in the *ada* gene decreased cell survival only slightly (Figure 6A), thereby confirming the results of the drop test. Importantly, the lack of AlkA protein was extremely harmful: after 5 min of treatment with 20 mM MMS the survival of *P. putida* alkA‾ mutant decreased already to 10%, and after 10 min of treatment to less than 1%. Similar survival dynamics appeared in the case of the ada‾alkA‾ double mutant. The ada‾alkA‾alkB‾ triple mutant was the most sensitive to MMS: after 15 min of MMS treatment only 0.01% cells survived (Figure 6A). The absence of AlkA showed the strongest effect on the survival of MNNG-treated *P. putida*, whereas the absence of AlkB increased the sensitivity of *P. putida* cells to MNNG only in the absence of AlkA or Ada (Figure 6C), which confirms the results of the drop test (Figure 5). Thus, taken together, the results of the MMS and MNNG sensitivity studies suggested that among the three studied DNA alkyl repair enzymes, the AlkA is the major player in protecting *P. putida* against alkyl damage. The presence of AlkB is required only in the absence of other repair mechanisms.

Entirely different results of sensitivity to MMS and MNNG were obtained with *E. coli* strains. *E. coli* alkA‾ mutant, like the wild-type strain, was not sensitive to 15 min treatment with 20 mM MMS (Figure 6B), but the survival of the alkB‾ and ada‾ mutants decreased to 23 and 3%, respectively. Survival of double ada‾alkA‾ mutant decreased to the level of 2% under such conditions (Figure 6B).

### Ada protein is crucial for MMS/MNNG-induced mutagenesis in *P. putida* and *E. coli*

**Figure 7:**
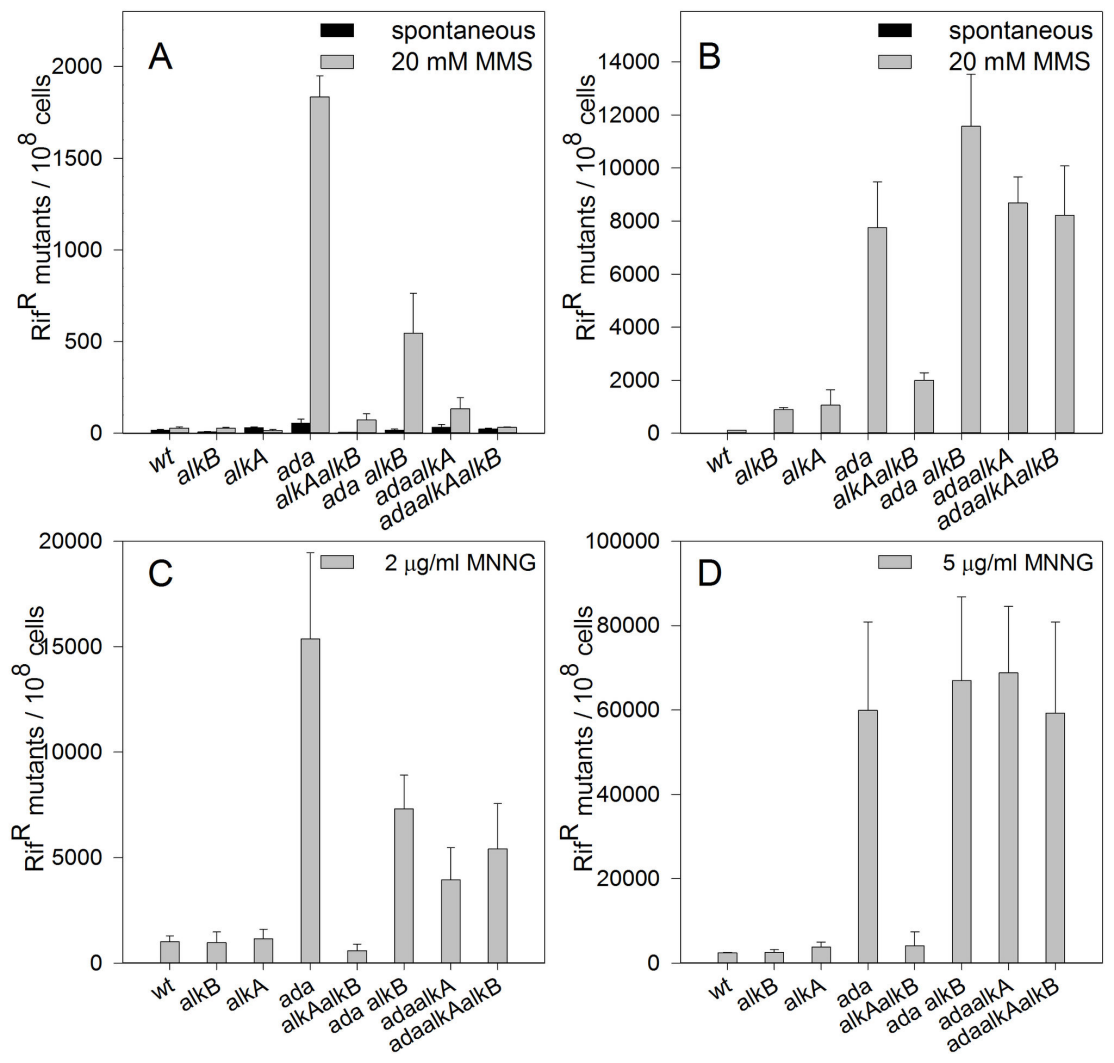
Frequency of MMS (A, B) or MNNG (C, D)-induced Rif^R^ mutations in *P. putida* (A, C) and *E. coli* (B, D) strains.

The results of survival assay after MMS/MNNG treatment indicated DNA repair proteins essential for protection of bacteria against cytotoxic action of alkylating agents. For *P. putida*, we have found AlkA protein to be crucial in this respect. On the other hand, the results of mutagenesis assay clearly indicate the key role of Ada protein in protecting *P. putida* cells against the mutagenic action of alkylating agents. *P. putida* strains defective in the *ada* gene showed the highest, among other mutants, level of MMS- and MNNG-induced Rif^R^ mutations, namely, about 1800 and 15,000 Rif^R^/10^8^ cells, respectively (Figure 7A and C). These results indicate that MMS/MNNG-induced *O* ^6^meG, if not directly demethylated by Ada methyltransferase, is a powerful source of mutations.

In *P. putida* ada‾ strains additionally mutated in *alkA* and/or *alkB* genes, the frequency of MNNG-induced Rif^R^ mutations was 2-3-fold lower than in the single ada‾ mutant (Figure 7C). In the case of *P. putida* alkA‾ mutant, the cytotoxic effect of MMS was so strong that the level of Rif^R^ mutations was not measurable under standard conditions. This observation concerns all the *alkA* mutants tested, namely, double ada‾alkA‾ and alkA‾alkB‾ and triple ada‾alkA‾alkB‾. Considering the strong cytotoxic effect of MMS on these mutants, we decided to shorten the time of MMS treatment to that resulting in about 30% of survival for each tested strain (note that in Figure 7A the frequency of mutations for *alkA* ^-^ strains represents the shorter time of MMS treatment). However, this approach did not influence the level of MMS-induced mutagenesis, indicating the key role of AlkA protein in protection of *P. putida* cells against cytotoxic but not mutagenic action of alkylating agents.

In *E. coli*, as in *P. putida*, the Ada protein was responsible for MNNG/MMS-induced mutagenesis. Thus, MNNG-treated bacteria mutated in the *ada* gene showed about 60,000 Rif^R^/10^8^ cells in comparison to 3000, 2000, and 4000 Rif ^R^/10^8^ cells in alkA‾, alkB‾, and alkA‾alkB‾ strains, respectively (Figure 7C and D). The frequency of MMS-induced Rif^R^ mutations in *E. coli* ada‾ strain was also the highest among single mutants (about 8000 *versus* 100 and 1000 Rif ^R^/10^8^ cells in alkA‾ and alkB‾ strains, respectively). However, in ada‾alkA‾, ada‾alkB‾, and ada‾alkA‾alkB‾ it remains on the high level of about 8700, 11,600 and 8200 Rif ^R^/10^8^ cells, respectively (Figure 7D). These results are dramatically different from those for the *P. putida* counterparts, e.g. in *E. coli* alkB‾ and alkA‾alkB‾ strains the frequencies of Rif^R^ mutations were, respectively, 30- and 36-fold higher in comparison to analogous *P. putida* mutants (Figure 7A and B). This indicates that, although EcAlkB and PpAlkB belong to the same subgroup of dioxygenases [23], their roles in the protection of *E. coli* and *P. putida* against alkylating agents are different. Similarly as in *P. putida* alkA‾, in *E. coli* alkA‾ strain, we observed an extremely low level of MMS-induced Rif^R^ mutations (about 55 Rif ^R^/10^8^ cells). However, in the presence of MMS, in contrast to *P. putida*, the *E. coli* alkA‾ mutant exhibited the same survival kinetics as the wild-type strain (Figure 6B).

**Figure 8:**
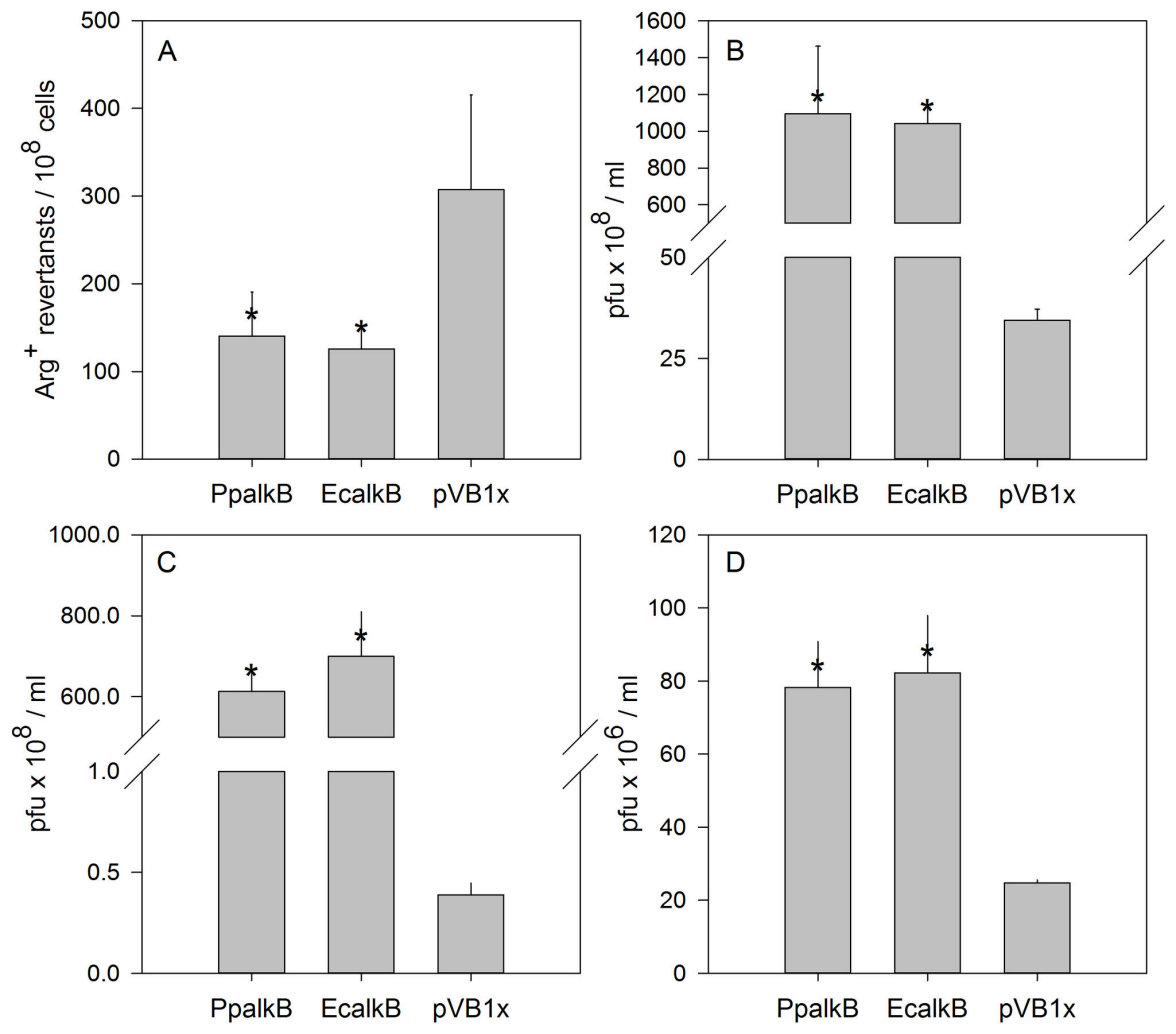
Complementation assay for EcAlkB protein. Frequency of MMS-induced Arg^+^ revertants in the *E*. *coli* alkB‾ strains harboring pVB1x plasmids expressing EcAlkB or the PpAlkB homolog (‘“empty”’ vector served as control) (A). Survival of MMS (B, D) or CAA (C) treated M13 (B, C) or MS2 (D) phages in the same strains.

It has been demonstrated that EcAlkB preferentially binds to single-stranded DNA (ssDNA) and repairs lesions produced in it [18]. AlkB dioxygenase also repairs methylation damage in RNA [32,33]. Therefore, PpAlkB ability to repair alkylation lesions in ssDNA and ssRNA was tested with the use of phage survival assays involving, respectively, M13 and MS2 phages. Two alkylating agents, the methylating MMS inducing 1meA and 3meC lesions (Figure 8B and D) and ethylating chloroacetaldehyde (CAA) inducing etheno adducts (Figure 8C) were tested. In *E. coli* alkB‾ strain, MMS-treated M13 phage survived at a level of about 3 × 10^9^ PFU/ml which is 30-fold lower than in *E. coli* alkB‾ but harboring the pVB1x plasmid expressing the Ec*alkB* gene (Figure 8B).

CAA-treated M13 phage survived almost 20,000-fold better in *E. coli* alkB‾ harboring pVB1x Ec*alkB* or pVB1x Pp*alkB* than in the strain bearing an “empty” pVB1x plasmid (about 7.2 × 10^8^ and 6.4 × 10^8^ *versus*. 3.7 × 10^4^ PFU/ml) (Figure 8C). However, the survival of MMS-treated RNA phage MS2 was only 4-fold elevated in *E. coli* alkB‾ expressing Ec*alkB* or pVB1x Pp*alkB* compared to cells bearing an empty plasmid (about 20 *versus* 80 × 10^6^ PFU/ml). These results indicate similar substrate specificity of EcAlkB and PpAlkB proteins.

### Substrate specificity of *P. putida* AlkB protein and optimal conditions for *in vitro* reaction

**Figure 9:**
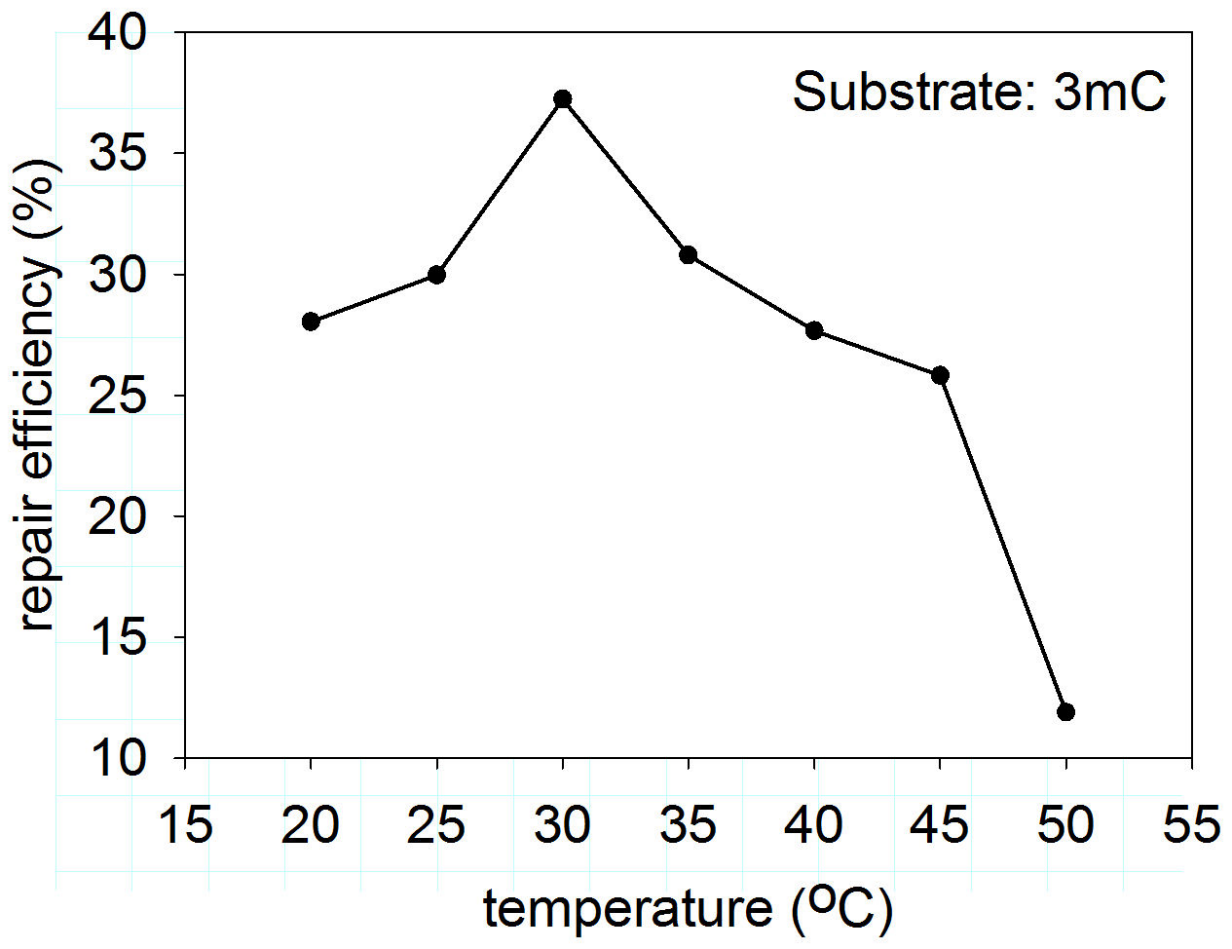
Dependence of 3meC repair by PpAlkB on the reaction temperature. Samples were assayed for extent of repair at marked temperature values.

**Figure 10:**
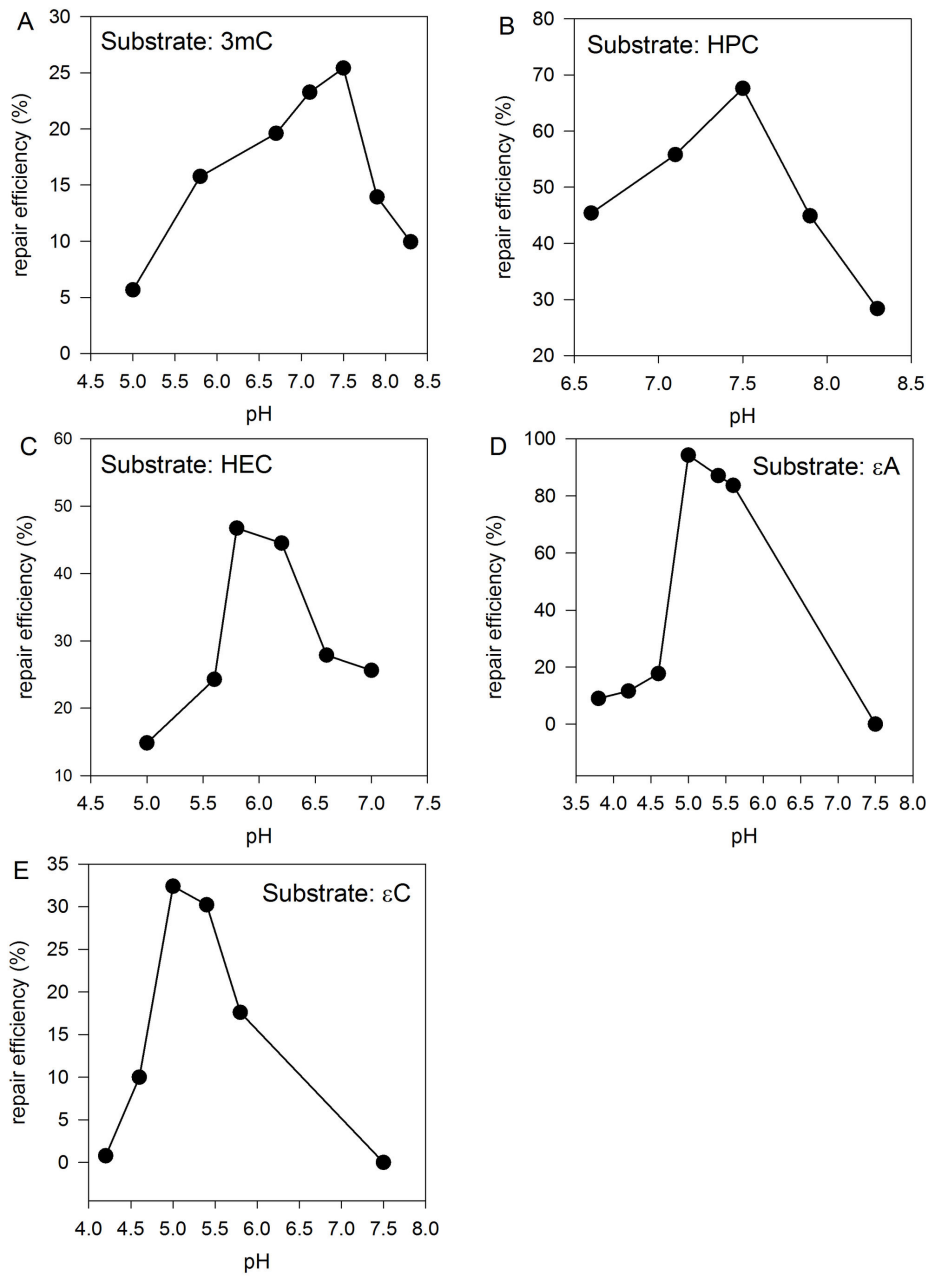
The pH dependence of repair rates of the studied adducts by PpAlkB protein. Samples were assayed for extent of repair at marked pH values at 30°C. The adducts and the conditions applied for their repair were as follows: 3meC (pKa 8.7; Ueda et al. (1963) J Amer Chem 85: 4024), 100 µM Fe(II) 5 pmol PpAlkB, 5 min reaction (A) HPC (pKa 8.6; Borys-Brzywczy et al. (2005) Acta Biochim Pol 52: 149), 100 µM Fe(II) 20 pmol PpAlkB, 15 min reaction (B); HEC (pKa 5.8; Krzyzosiak et al. (1979) Polish J Chem 53: 243), 1 mM Fe(II) 20 pmol PpAlkB, 15 min reaction (C) ;εA (pKa 3.9; Secrist et al. (1972) Biochemistry 11: 3499), 3mM Fe(II) 40 pmol PpAlkB, 15 min reaction (D); and εC (pKa 3.7; Krzyzosiak et al. (1979) Polish J Chem 53: 243), 50 µM Fe(II) 80 pmol PpAlkB, 15 min reaction (E).

**Figure 11:**
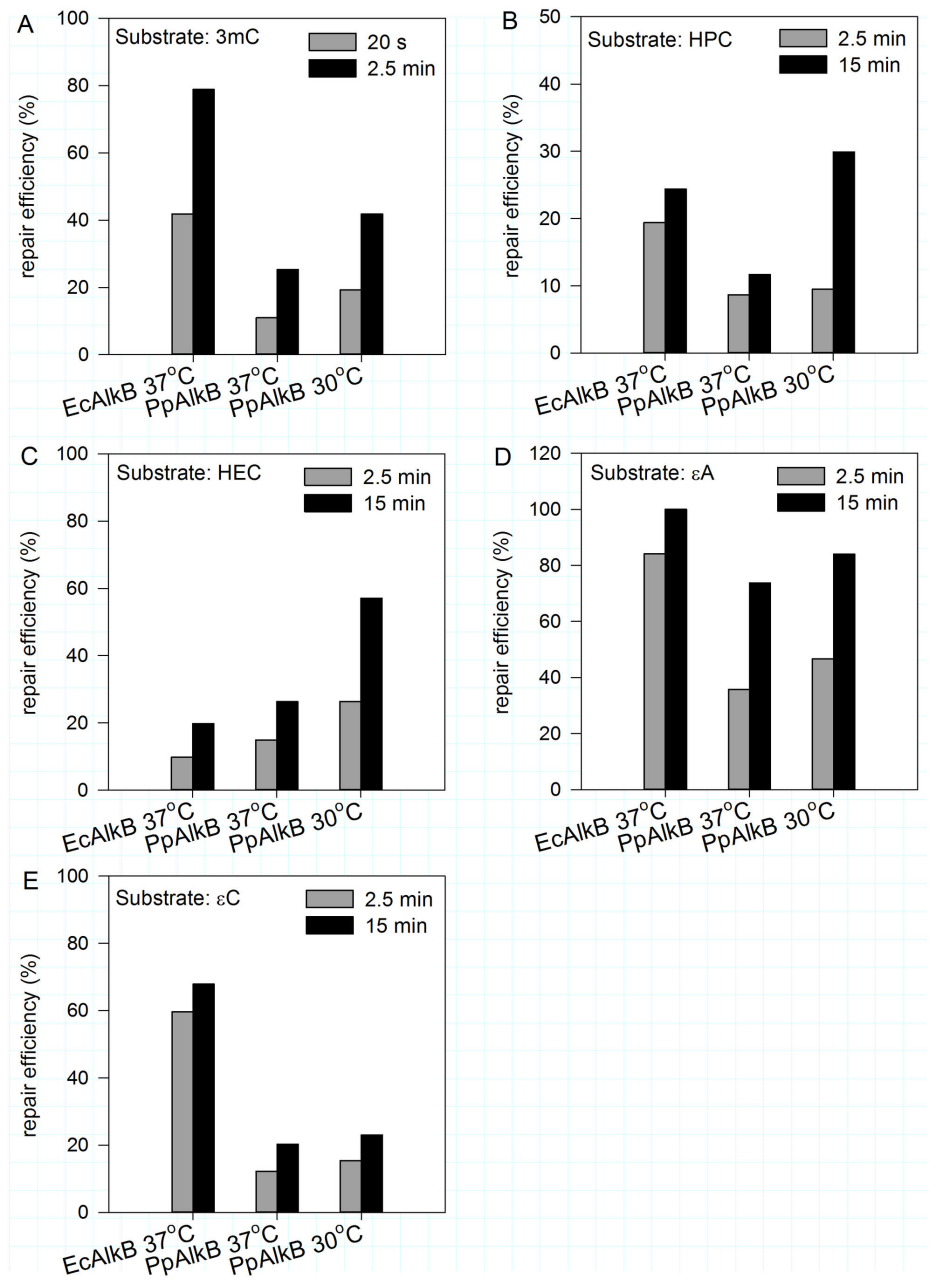
The time course of repair of the studied adducts at the pH optimal for each substrate. Comparison of repair efficiency between EcAlkB at 37°C and PpAlkB at 30 and 37°C. Reactions for each substrate (0.5 nmol) were performed in parallel and differ only in the protein added. The adducts and the conditions applied for their repair were as follows: 3meC, pH 7.5, 100 µM Fe(II), 5 pmol of the studied enzyme (A); HPC pH 7.5, 100 µM Fe(II), 10 pmol of the studied enzyme (B); HEC pH 5.8, 1 mM Fe(II), 10 pmol of the studied enzyme (C); εA pH 5.0 for PpAlkB and 4.6 for EcAlkB, 3 mM Fe(II) 40 pmol of the studied enzyme (D); and εC pH 5.0 for PpAlkB and 4.6 for EcAlkB, 5 mM Fe(II), 80 pmol of the studied enzyme (E).

For more precise characterization of PpAlkB, we overexpressed and purified this protein in a heterologous system. Pentamers containing modified bases: 3meC, HPC, HEC, εC or εA were studied as potential substrates for PpAlkB. Using HPLC to separate the modified and unmodified (repaired) oligomers, we found that all the modifications were repaired. Since *P. putida* is a soil bacterium, the PpAlkB tolerance to temperature was tested. As shown in Figure 9, the optimal temperature for 3meC repair was 30°C. Optimal pH for 3meC and HPC repair was 7.5, for HEC -5.8, for εA and εC – pH 5.0 (see Figure 10 for details). This is in agreement with our previous findings concerning pH dependence of adduct repair rates by EcAlkB [22]; however, in case of PpAlkB, εA and εC were best repaired at higher pH in comparison to EcAlkB (see Discussion). Parallel experiments at optimal pH for each adduct compared Pp and Ec AlkB repair efficiencies. Reactions conducted at various temperatures confirmed that PpAlkB is more active at 30°C than 37°C, suggesting that, in spite of several noticeable differences, the adducts studied are repaired by both dioxygenases with comparable efficiency (Figure 11). Nevertheless, it is worth noting that 3meC, the best substrate for both enzymes, was repaired by PpAlkB about twice less efficiently.

## Discussion

### The role of Ada response in protection of *P. putida* cells against alkylating agents

The aim of this work was to assign the role of Ada, AlkA, and AlkB proteins to the repair of alkylation lesions in *P. putida* DNA. *In silico* and *in vivo* studies disclosed fundamental differences between *P. putida* and *E. coli* Ada operon organization and AlkA protein structure as well as different reactions of these bacteria to alkylating agent treatment. Ada operon of *P. putida* encodes for AlkA and Ada, but not for AlkB, whereas in *E. coli ada* and *alkB* comprise one operon and *alkA* is situated separately, although stays under control of *ada* promoter. The *P. putida* genome encodes for three additional repair enzymes: PpTag2, PpAlkA2 and Aag (Figure 1). However, *alkA2* is not equally distributed among *P. putida* isolates. For example, *P. putida* strain KT2440, used as a model organism in this study, lacks this gene. Among the 11 analyzed *P. putida* genomes only four carry *alkA2*.

*P. putida* and *E. coli* strains mutated in the *ada* gene are diametrically opposed in response to alkylating agents, MMS and MNNG (see Table 1 and Results). On the basis of this sensitivity three groups of mutants have been distinguished: insensitive, moderately and highly sensitive. Deletion of the *alkA* gene in *P. putida* results in extreme sensitivity to both alkylating agents. In contrast, the *E. coli* alkA‾ mutant is not more sensitive to them than the wild-type strain. These observations indicate the crucial role of AlkA protein (*alkA*-encoded 3meA DNA glycosylase II) in protecting *P. putida* cells against the cytotoxicity of the alkylating agents. However, mutagenicity test revealed that AlkA did not protect *P. putida* cells against the mutagenic action of MMS/MNNG. Instead, the Ada protein fulfills an anti-mutagenic function (*P. putida* ada‾ mutants showed an increased frequency of MMS/MNNG-induced Rif^R^ mutations, Figure 7A and C). In *E. coli* alkB‾ strain, MMS-induced mutagenesis measured in *argE3*→Arg^+^ reversion system increased about 8-fold in comparison to the wild-type strain [34]. Moreover, the presence of DNA polymerase V (PolV) is indispensable to process 1meA/3meC lesions in an error-prone manner [35]. The lack of this type of response to alkylating agents in *P. putida* alkB‾ is probably due to different importance of SOS responses and PolV-directed DNA repair in these two bacterial species. Importantly, the majority of bacteria, including pseudomonads, lack chromosomal *umuC* and *umuD* genes encoding PolV but carry instead a multiple gene cassette encoding DnaE2, a second copy of the α subunit of DNA polymerase III, and ImuB, a protein related to the Y-family of DNA polymerases [36-38]. Similarly to *umuDC*, these genes are induced by DNA damage [37]. The fact that the presence of *dnaE2* and *imuB* genes coincides with the lack of *umuDC* genes in the bacterial genome has suggested that in these species the gene cassette functionally replaces PolV in translesion synthesis [38]. Current studies in our laboratories might elucidate the role of DnaE2 in *P. putida* tolerance to DNA alkyl damage.

**Table 1:** Sensitivity of *P. putida* and *E. coli* mutants to MNNG and MMS exposure.

Sensitivity	MNNG		MMS	
	*P. putida*	*E. coli*	*P. putida*	*E. coli*
Survival like wild-type	*alkB*	*alkA*	*alkB*	*alkA*
		*alkB*		
		*tag*		
Moderately sensitive	*ada*	*ada*	*ada*	*alkB*
	*ada alkB*		*ada alkB*	*ada alkB*
Very sensitive	*alkA alkB*	*alkA alkB*	*alkA alkB*	*alkA alkB*
	*alkA*	*ada alkA*	*alkA*	*ada*
	*ada alkA*	*ada alkB*	*ada alkA*	*ada alkA*
	*ada alkA alkB*	*ada alkA alkB*	*ada alkA alkB*	*ada alkA alkB*

The comparison of sequences of AlkA from *P. putida*, *E. coli*, and *D. radiodurans* (DrAlkA) revealed great similarity of PpAlkA to DrAlkA but not to EcAlkA (Figure 3). Both enzymes consist of two helical domains separated by a wide DNA-binding cleft but devoid of the third N-terminal α/β domain present in EcAlkA [12]; hence, the activity towards 3meC and 1meA, the most efficiently repaired canonical substrates of AlkB dioxygenase, characterizes both PpAlkA and DrAlkA enzymes. Of noted, species lacking the AlkB homolog developed AlkA proteins capable of repair of its canonical substrates [13,30]. Thus, it can be proposed that *P. putida* (at least KT2440) is on the developmental path that would eventually results in complete lack of the *alkB* gene.

### The *alkA* but Not *alkB* Gene Is Induced by the Ada Response in *P. putida*

The Ada response has been comprehensively studied in *Enterobacteria* [39,40]. In spite of the high conservation of its function, the Ada regulon is diversely organized [41,42], as confirmed by our results. We have shown that in *P. putida* the level of transcription from the promoter located upstream of *alkA* is induced significantly after exposure to MMS (Figure 4). Its expression elevated about 60-fold, whereas in *E. coli* the increase was only 10-fold [39]. In contrast, the *P. putida alkB* gene was not induced after MMS treatment although it exhibited a constantly significant level of expression, regardless of the presence of the alkylating agent (Figure 4).

In *E. coli*, two promoter regions, *ada* and *alkA*, show highly similar nucleotide fragments responsible for the interaction with the Ada protein, respectively, AAAGCGCA and AAAGCAA [43,44]. The *alkA* promoter region of *P. putida* seems to preserve the *E. coli* Ada box core in the form of ATT(N) _6_GCAA (Figure 3). Additionally, the consensus sequence of this promoter lies from -35/-10 boxes in the distance similar to that of *E. coli ada* and *aidB* promoters, which implies the interaction with RNA polymerase through the CTD domain of Ada protein [45]. The *ada* promoter consensus is highly conserved among bacteria. In *Salmonella typhimurium*, it is in the form of GAATTAAAACGCA although the Ada protein shows lower transcription factor activity than the *E. coli* protein [42]. On the other hand, the *ada* and *aidB* promoters of *E. coli* interact with the Ada protein through the core of AAT(N) _6_GCAA sequence [46], which is perfectly preserved in the putative *aidB* promoter of *P. putida* (Figure 4).

Motif identification shed more light on the Ada box occurrence. He and co-workers [30] showed that the promoter structure required for Ada binding in *E. coli* consists of box A (AAT) and B (GCAA), both being responsible for specific sequence interactions. Two Arg residues, 45 and 71, of *E. coli* Ada protein form a net of hydrogen bonding with thymine residues. The A box in *P. putida alkA* promoter can be considered as conserved in the form of (-79)-5’-ATT-3’-(-77), allowing for interaction with the Ada protein (Figure 4). The well conserved B box is separated by 6 nucleotides. There is no doubt that in *P. putida* strains Ada boxes are preserved in promoter regions of *alkA* and *aidB* genes with three A/T base pair in A box and GCAA in B box (Figure 3).

The Ada box cannot be identified with high fidelity in the promoter regions of the *alkB* gene in *P. putida* species (it was found only in three of them with *P*-value below 0.01); nevertheless, all of these promoter regions contain putative -35/-10 boxes (Figure 3). This suggests that the basal expression of the *alkB* genes in *P. putida* does not depend on the Ada regulator (the basal expression was also increased in *P. putida* ada‾ strain and in *E. coli*) or any other transcriptional factor.

It has been established that in *E. coli* basal expression of the *ada* gene results in about 1-2 molecules of Ada [41]. This seems not to be true for *P. putida* Ada regulon since the promoter for *alkA-ada* operon producing the Ada protein did not show any basal expression. Thus, at the moment, the initiation of Ada response in *P. putida* cannot be explained.

Although the Ada motifs are almost identical in promoter regions of Pp*alkA* and Ec*ada*, exchanging these motifs between the two species did not result in proper functioning in both backgrounds. This surprising phenomenon can be explained as follows: i) the spaces between Ada and -35 boxes are different in these two promoters potentially affecting the interaction with RNA polymerases; ii) in spite of perfect conservation of *P. putida* Ada residues responsible for specific interaction with the Ada box, tiny differences between *E. coli* and *P. putida* Ada boxes prevent cross-species GFP induction. The PpAda protein seems to rely less on the interaction with the A box since removal of this box and additional four bases upstream of it still resulted in slight induction by the methylating agents (Figure 4). It appears as if even one A base can establish some interaction with PpAda, provided that the B box is present.

The Pp*alkA* and Pp*GcdH* promoters could be considered to be strongly regulated by the Ada protein since C is the base 5’-adjacent to the B box. Landini and Volkert [46] have shown that the difference in this position between *ada* and *alkA/aidB E. coli* promoters, consisting of C and A base, respectively, renders the Ada protein with about 10-fold higher affinity for the *ada* promoter.

The majority of earlier induction experiments on the *E. coli* Ada regulon genes were conducted using episomal multicopy vectors. A similar approach was undertaken in the present study on *P. putida* Ada regulon. Nevertheless, these experiments should be considered preliminary. A more reliable and trustworthy experimental approach would be to test the induction from single copy chromosome constructs. To summarize, the results presented in this study indicate that the *P. putida alkA-ada* operon is strongly induced in the course of Ada response to alkylating agents, interacting not only with *E. coli* AlkA substrates but also with 1meA and 3meC, considered to be the most efficiently repaired AlkB substrates. Moreover, the promoter sequence of *P. putida alkB* gene is active at moderate levels independently of the presence of any exogenous alkylating agent, indicating that, unlike in *E. coli*, in *P. putida* the AlkB protein is expressed constitutively and is not under control of the Ada response.

### Characterization of *P. putida* AlkB protein – substrate specificity and optimal conditions for *in vitro* reaction

*E. coli* AlkB protein has been described as a single-protein repair system protecting cellular DNA and RNA against the cytotoxic and mutagenic activities of alkylating agents, tumorigenic chemicals and also those used in anticancer therapy. EcAlkB homologs exist in almost all organisms. Our *in silico* analysis allowed identification of 1943 sequences of different AlkB family members and eight novel subfamilies of AlkB homologs [23]. In the present work we characterized the AlkB protein of *P. putida* (PpAlkB) to compare its activity and substrate specificity to that of *E. coli* AlkB.

Recently, we have characterized the substrate specificity and some aspects of molecular mechanism of action of *E. coli* AlkB, and established that EcAlkB repair efficiency is pH- and Fe(II)-concentration dependent, which correlates with the substrate pK_a_. The experimental data and molecular modeling revealed that *E. coli* AlkB dioxygenase preferentially recognizes and repairs protonated substrates. Crucial for this recognition is the negatively charged carboxylic group of Asp135 side chain in the enzyme active center [22]. *E. coli* and *P. putida* AlkB sequences alignment showed that the organization of active centers is identical, including Asp135 (Asp136 in the case of *P. putida* KT2440 strain). The screening experiments performed here confirmed all of our previous findings. For 3meC, HPC, and HEC substrates, the optimal pH of the repair reaction was about one unit below their pK_a_ where the adducts existed in the cationic form (Figure 11). For adducts with low pK_a_ values, εA and εC, the optimal pH is a compromise between the substrate protonation and other factors determining the efficiency of repair (discussed in details in [22]). The optimal pH for εA and εC repair by PpAlkB is 5.0, slightly higher than the 4.6 for EcAlkB [22], suggesting that the former may be more susceptible to unfolding under acidic conditions. Similarly as in the case of EcAlkB, the etheno adducts were less efficiently repaired by PpAlkB than the hydroxyethano and hydroxypropano adducts. The presence of AlkB dioxygenase in *P. putida* cells, in spite of the extended substrate specificity of *P. putida* AlkA, suggests PpAlkB role in repair of still other cytotoxic/mutagenic substrates.

In conclusion, the results presented here indicate different organization of *P. putida* and *E. coli* Ada response and also quite different functions of the individual proteins induced by Ada. The AlkA protein plays a crucial role in protecting *P. putida* cells against cytotoxic but not mutagenic action of alkylating agents, whereas the Ada protein shows a strong anti-mutagenic function. We have not been able to connect any of these functions to PpAlkB, in spite of its enzymatic activity identical to that of EcAlkB. Ada regulon organization and promoter activity studies indicate, apart from the Ada response, constitutive expression of PpAlkB. This discovery, in spite of the similarity of *P. putida* and *E. coli* AlkB sequences, shows a different role of PpAlkB in protecting *P. putida* cells against alkylating agents of endo- or exogenous origin. On the basis of analogy to organization of anti-alkylating agent defense system in *E. coli*, we suppose that PpAlkB plays its role together with constitutively expressed Tag and Ogt proteins. Regulation of *P. putida*, but not of *E. coli*, AlkB resembles the situation in higher organisms where ALKBHs are widely expressed in normal tissues. Therefore, *P. putida* can serve as a model organism for establishing novel ALKBHs functions in higher eukaryotes. The regulation of ALKBHs expression is essential for understanding the basis of this system since upregulation of ALKBHs underlies tumor formation and its resistance to alkylating chemotherapy.

## Materials and Methods

### Bacterial strains, plasmids, and media

The bacterial strains and plasmids used in this study are listed in Table 2. Liquid media used were Luria-Bertani broth (LB) [47], M9 minimal medium [48], and *E medium* composed of C salts [49], glucose (0.5%), casamino acids (0.2%), and thiamine (10 µg/ml). The solid media contained 1.5% Difco agar. LCA medium (1% trypton, 0.5% yeast extract, 1% NaCl, 0.25% MgSO_4_ × 7H_2_O, 2.5 mM CaCl_2_) was solidified with Difco agar at 0.6% [47]. Antibiotics were added at the following concentrations: rifampicin at 100 µg/ml, carbenicillin at 100 µg/ml for *E. coli* and 2 mg/ml for *P. putida*, kanamycin at 50 µg/ml, and gentamicin at 10 µg/ml. Bacteria were grown on agar plates or in liquid cultures with shaking (250 rpm), *E. coli* at 37°C, and *P. putida* at 30°C. *E. coli* was electrotransformed as described by Sharma and Schimke [50] and *P. putida* as in ref [51]. . *E. coli* strains DH5α (Invitrogen), and CC118 λpir [52] were used for DNA cloning, and HB101 [53] as a host for helper plasmid pRK2013 [54], necessary for mobilization of non-conjugative plasmids.

**Table 2:** Bacterial strains and plasmids used in this study.

**Strain or plasmid**	**Genotype or construction**	**Source or reference**
Strains		
*E. coli*		
DH5α	*supE44 ΔlacU169 (Φ80 lacZΔM15) recA1 endA1 hsdR17 thi-1 gyrA96 relA1*	Invitrogen
HB101	*subE44 subF58 hsdS3 (r_B_^-^ m_B_^-^) recA13 ara-14 proA2 lacY1 galK2 rpsL20*	[53]
	*xyl-5 mtl-1*	
CC118 λpir	*Δ(ara-leu) araD ΔlacX74 galE galK phoA20 thi-1 rpsE rpoB argE (Am)*	[52]
	*recA1 λpir phage lysogen*	
BL21(DE3)pLysS	*F^-^ ompT gal dcm lon hsdS_B_(r_B_^-^ m_B_^-^) λ(DE3) pLysS(cm^R^)*	Invitrogen
AB1157	*thr-1, araC14, leuB6(Am), Δ(gpt-proA)62, lacY1, tsx-33, qsr’-0, glnV44(AS)*	[71]
	*galK2(Oc), LAM-, Rac-0, hisG4(Oc), rfbC1, mgl-51, rpoS396(Am) rpsL31(strR),*	
	*kdgK51, xylA5, mtl-1, argE3(Oc), thi-1*	
AB1157alkA	As AB1157 but *∆alkA::km*	This study
DM12	As AB1157 but *∆alkB::km*	[23]
AB1157ada	As AB1157 but *ada10::Tn10* ; constructed by P1 phage transduction	This study
BS87	As AB1157 but *∆alkB::ap*	[72]
BS87alkA	BS87 but *∆alkA::km*; constructed by P1 phage transduction	This study
AB1157alkAada	As AB1157 but *∆alkA::km; ada10::Tn10*	This study
AB1157alkBada	As AB1157 but *∆alkB::km; ada10::Tn10*	This study
BS87alkAada	As BS87 but *∆alkA::km; ada10::Tn10*	This study
UC978	*araD8l* Δ(*uvrB-bio*) *∆ogt1::km ada10::Tn10*; used as a donor of *ada10::Tn10*	[73]
*P. putida*		
PaW85	Wild-type, isogenic to KT2440	[55,56]
PaWAlkB	PaW85, *∆alkB::gm*	This study
PaWAda	PaW85, *Δada::km*	This study
PaWAlkA	PaW85, *∆alkA*	This study
PaWAdaAlkB	PaW85, *Δada::km*; Δ*alkB*::*gm*	This study
PaWAlkAAlkB	PaW85, ∆*alkA*; Δ*alkB*::*gm*	This study
PaWAlkAAda	PaW85, ∆*alkA*; ∆*ada::km*	This study
PaWAlkAAdaAlkB	PaW85, Δ*alkA*; ∆*ada::km*; Δ*alkB*::*gm*	This study
Plasmids		
pBluescript KS (+)	Cloning vector (Ap^r^)	Stratagene
pUTmini-Tn*5* Km2	Delivery plasmid for mini-Tn*5* Km2 (Ap^r^ Km^r^)	[59]
pBK-miniTn*7*-ΩGm	pUC19-based delivery plasmid for miniTn*7*-ΩGm (Gm^r^, Ap^r^)	[58]
pGP704 L	Delivery plasmid for homologous recombination (Ap^r^)	[57]
pRK2013	Helper plasmid for conjugal transfer of pGP704 L (Km^r^)	[54]
pEMG	Cloning vector (Km^r^), *ori*R6K, *lacZ*α with two flanking I-SceI sites	[61]
pSW (I-SceI)	The I-SceI-expression plasmid (Ap^r^)	[62]
pKSalkB	pBluescript KS (+) containing the PCR-amplified sequence of the *alkB* gene	This study
	in SacI and Acc65I restriction sites	
pKSΔalkB::gm	*alkB* in pKSalkB is interrupted with the Gm^r^ gene from pBK-miniTn7-ΩGm	This study
	by replacing SacII and HindIII-generated fragment from *alkB* by the Gm^r^ gene	
pGP704ΔalkB::gm	pGP704 L with SacI-Acc65I fragment of *ΔalkB*::*gm* from pKS∆alkB::gm	This study
	in vector plasmid opened with the same restrictases	
pKSada	pBluescript KS(+) containing the PCR-amplified sequence of the *ada* gene in SacI	This study
	and XbaI restriction sites	
pKSΔada::km	*ada* in pKSada is interrupted with the Km^r^ gene from pUTmini-Tn*5* Km2 by	This study
	replacing Cfr42I and NcoI-generated fragment from *ada* by the Km^r^ gene	
pGP704Δada::km	pGP704 L with SacI-XbaI fragment of *Δada*::*km* from pKSΔada::km in vector	This study
	plasmid opened with the same restrictases	
pEMGalkA	pEMG containing PCR-amplified sequence of *alkA* in SacI and XbaI sites	This study
pVB1x	A low copy number vector (~6 copies/cell) with a toluic acid inducible	Vectron Biosolutions
	promoter (Ap^r^)	
pVB1x:*EcalkB*	As pVB1x but expressing EcalkB	This study
pVB1x:*PpalkB*	As pVB1x but expressing PpalkB	This study
pET28a(+)	Expression vector (Km^r^)	Invitrogen
pET28a::*PpalkB*	As pET28a(+) but expressing PpalkB	This study
pPROBE-NT	Promoterless vector with gfp reporter (Km^r^)	[63]
pPROBE-AT	Promoterless vector with gfp reporter (Ap^r^)	[63]
pPROBE-NT:*alkAp*	As pPROBE-NT with *P. putida alkA* promoter (-100, -1)	This study
pPROBE-NT:*alkBp*	As pPROBE-NT with *P. putida alkB* promoter (-100, -1)	This study
pPROBE-NT:*alkBp*Δ(-68,-18)	As pPROBE-NT with *P. putida alkB* promoter (-100, -1)	This study
	with deletion of (-68,-18) nucleotides	
pPROBE-NT:*adap*	As pPROBE-NT with *P. putida ada* promoter (-100, -1)	This study
pPROBE-NT:Ec*adap*	As pPROBE-NT with *E. coli ada* promoter (-100, -1)	This study
pPROBE-AT:*alkAp*	As pPROBE-AT with *P. putida alkA* promoter (-100, -1)	This study
pPROBE-AT:*alkBp*	As pPROBE-AT with *P. putida alkB* promoter (-100, -1)	This study
pPROBE-KT:*kan*p	As pPROBE-KT but with kanamycin resistance gene promoter	Prof. S. E. Lindow
		Berkeley, California

## Construction of *P. putida* Ada and AlkB-Deficient Strains

The sequences of *alkB* gene encoding for oxidative demethylase (PP_3400) and *ada* gene encoding for methyltransferase (PP_0706) of *P. putida* strain KT2440 were obtained from The Comprehensive Microbial Resource website of the J. Craig Venter Institute (http://cmr.jcvi.org). These genes were amplified by PCR from the genomic DNA of *P. putida* strain PaW85 [55] which is isogenic to KT2440 [56]. Thereafter, the internal sequences of the amplified genes were replaced with antibiotic resistance marker genes and the DNA fragments carrying the antibiotic resistance gene and sequences from 5´- and 3´- ends of the *alkB* or *ada* gene were inserted into plasmid pGP704L [57] unable to replicate in hosts other than *E. coli* CC118λpir [52]. The wild-type sequences of the *alkB* and *ada* genes present in the chromosome of *P. putida* strain PaW85 were replaced with the interrupted ones by homologous recombination. Derivatives of the plasmid pGP704L carrying replacement cassettes were conjugatively transferred into *P. putida* PaW85 using helper plasmid pRK2013 [54]. The integration of whole delivery plasmid into a target site was excluded by testing the transconjugants for resistance to carbenicillin (only those unable to grow in the presence of 3 mg/ml carbenicillin were considered to be true recombinants, generated as a result of double recombination events). PaW85 derivatives with the desired deletions were verified by PCR analysis.

For the construction of *P. putida* strain lacking *alkB* (PaWAlkB), the 648-bp *alkB* gene was amplified by PCR from the genomic DNA of *P. putida* with primers ppalkBFwSacuus and ppalkBRevAccuus (see Table S1). The amplified 1275-bp DNA fragment containing the *alkB* gene was cleaved with SacI and Acc65I restriction enzymes (restriction sites in primers are underlined) and inserted into the vector plasmid pBluescript KS (+) opened with the same restrictases to obtain plasmid pKSalkB. The Gm^r^ gene was amplified by PCR from plasmid pBK-miniTn7-ΩGm [58] using primers GmY and GmA. The 771-bp PCR fragment containing the Gm^r^ gene was subsequently inserted into plasmid pKSalkB cleaved with SacII- and HindIII, and replaced the 850-bp fragment in the *alkB* gene in plasmid pKSalkB to obtain plasmid pKS∆alkB::gm. The SacII and HindIII-ends were blunt-ended before ligation. The SacI- and Acc65I-generated DNA fragment containing the *∆alkB::gm* sequence from plasmid pKS∆alkB::gm was inserted into plasmid pGP704 L cleaved with the same enzymes. The resulting plasmid pGP704∆alkB::gm was selected in *E. coli* strain CC118 λpir. The PaW85 *ΔalkB::gm* knockout mutant PaWAlkB was verified by PCR analysis using primers ppalkBlookusuus and GmA.

For the construction of *P. putida* strain lacking *ada* (PaWAda), the 1053-bp *ada* gene was amplified by PCR from the genomic DNA of *P. putida* with primers ppadaFwSac and ppadaRevXba. The amplified 1098 bp DNA fragment containing the *ada* gene was cleaved with SacI and XbaI restriction enzymes and inserted into the vector plasmid pBluescript KS (+) opened with the same restrictases to obtain plasmid pKSada. The Km^r^ gene was amplified by PCR from plasmid pUTmini-Tn5 Km2 [59] by using the primer KmSac [60]. The Ecl136II-cleaved DNA fragment containing the Km^r^ gene was used to replace the Cfr42I- and NcoI-generated 734-bp fragment in the *ada* gene in plasmid pKSada to obtain plasmid pKS∆ada::km. The Cfr42I and NcoI-ends were blunt-ended before ligation. The SacI- and XbaI-generated DNA fragment containing the *Δada::km* sequence from plasmid pKS∆ada::km was inserted into plasmid pGP704 L cleaved with the same enzymes. The resulting plasmid pGP704∆ada::km was selected in *E. coli* strain CC118 λpir. The PaW85 *Δada::km* knockout mutant PaWAda was verified by PCR analysis using primers ppadalookus and KmH.

### Construction of *P. putida* AlkA-deficient strain

For the construction of *P. putida* strain lacking the sequence of the *alkA* gene coding for DNA 3meA glycosylase, we used an adaptation of the protocol for *P. putida* [61]. The *alkA* gene (PP_0705) was amplified by PCR from the genomic DNA of *P. putida*. Two pairs of PCR primers that each span 500 bp of either upstream or downstream sequence of the *alkA* gene were designated ppAlkA1SacIFw and ppAlkA1Rev, and ppAlkA2Fw and ppAlkA2XbaIRev, respectively. SacI and XbaI restriction sites were included in the primers. Firstly, the 573 bp PCR product generated by primers ppAlkA1SacIFw and ppAlkA1Rev, and the 623 bp PCR product generated by primers ppAlkA2Fw and ppAlkA2XbaIRev were prepared. Then, the second PCR reaction was performed with 1 µl of each of the reaction products of the first PCR as a template using primers ppAlkA1SacIFw and ppAlkA2XbaIRev. After purification the second PCR product was cleaved with SacI and XbaI enzymes and inserted into the vector plasmid pEMG [61] cut with the same restrictases to obtain plasmid pEMGalkA selected in *E. coli* strain CC118 λpir. Plasmid pEMGalkA was then electroporated into the recipient strain of *P. putida* PaW85. Several colonies were then streaked and primers ppAlkA1SacIFw and ppAlkA2XbaIRev were used to check for cointegration. The plasmid pSW (I-SceI) [62] was electroporated into the *P. putida* PaW85 strain carrying the cointegrated *alkA* gene The presence of pSW (I-SceI) plasmid was verified by PCR analysis using primers pSW-F and pSW-R [61]. To obtain separate colonies several *P. putida* clones carrying the *alkA* gene and pSW (I-SceI) were streaked onto plates containing carbenicillin 3 mg/ml. To check for loss of the cointegrated plasmid pEMGalkA, single colonies were then streaked onto LB and LB kanamycin plates. Selected kanamycin sensitive clones were further analyzed by PCR using primers ppAlkA1SacIFw and ppAlkA2XbaIRev. In the next step, the potential clone bearing the *alkA* deletion was grown overnight with shaking, in LB medium without antibiotics, and dilutions in LB media were plated to obtain separate colonies. Several colonies were then restreaked in LB and LB carbenicillin media. The loss of the pSW (I-SceI) plasmid in carbenicillin sensitive clones was checked by PCR analysis using primers pSW-F and pSW-R. The PaW85 *ΔalkA* knockout mutant PaWAlkA was verified by PCR analysis using primers ppAlkAKI and ppAlkAKII.

### Construction of *P. putida* double and triple mutants

To isolate double mutants of PaW85 lacking both AlkB function and either alkyltransferase Ada (strain PaWAdaAlkB) or 3meA glycosylase activity encoded by the *alkA* gene (strain PaWAlkAAlkB), the existing PaWAda and PaWAlkA strains were used as recipients. *∆alkB::gm* sequence from the plasmid pGP704∆alkB::gm was used to delete the original *alkB* sequence from these strains. To verify that the sequence of the original *alkB* gene was replaced by the modified one, we examined all the constructed double knockout strains by PCR analysis using the primers described above. To isolate double mutant of PaW85 lacking both the AlkA and Ada functions (strain PaWAlkAAda), the existing PaWAda strain was used as a recipient and the *alkA* gene was deleted as described above. To isolate the triple mutant of PaW85 lacking AlkA, Ada and AlkB function (strain PaWAlkAAdaAlkB), the existing PaWAdaAlkB strain was used as a recipient, and the *alkA* gene was deleted using the same method as for construction of PaWAlkA.

### Plasmid construction for PpAlkB overexpression/complementation and for promoter induction assays

The pET28(a) and pVB1x plasmids were used to insert *P. putida alkB* gene PCR product produced with Pputup/Pputdn primers (Table S2). Selected primers allowed for the addition of NdeI and XhoI restriction sites to the PCR product and subsequent digestion and ligation with plasmids digested in the same way.

The pPROBE-NT plasmid [63] allowing the insertion of the promoter sequence upstream of the gene coding for the GFP protein was used to monitor the activity of *P. putida alkA*, *alkB*, and *ada* promoters. The PCR products obtained with PpalkAH1/PpalkAdE, PpalkBH1/PpalkBdE, PpadauH1/PpadadnE primer pairs on *P. putida* genomic DNA allowed cleavage with HindIII and EcoRI and subsequent insertion into pPROBE-NT. The DNA sequences locating at the positions -100 to -1 from the ATG start codons of *alkB*, *ada* and *alkA* genes were inserted into the promoter probe vector to obtain the plasmids pPROBE-NT:*alkB*p (*alkBp*), pPROBE-NT:*ada*p (*adap*), and pPROBE-NT:*alkA*p (*alkAp*). Additionally, the promoter sequence for the *alkB* gene with region deleted for -68 to -18 nucleotides was used to confirm the putative sequences (pPROBE-NT:*alkB*pΔ). To investigate the induction of *alkA* and *alkB* promoters in *P. putida* ada*‾* strain which harbors kanamycin resistance, the pPROBE-AT vector (carbenicillin resistance) was used in place of pPROBE-NT. Also the pPROBE-NT construct with *E. coli ada* promoter (-100, -1) was prepared. The pPROBE-KT:*kan*p construct expressing GFP under the strong kanamycin resistance gene promoter was used to verify the experimental protocol [63]. Primers are listed in Table S2.

The deletion/mutation constructs of pPROBE-NT:*alkA*p were prepared by site directed mutagenesis with the following primers: A box del – AalkAup/AalkAdn; B box del – BalkAup/BalkAdn; AB boxes del – ABalkAup/ABalkAdn; A box mut – AmalkAup/AmalkAdn; AA box del – AaalkAup/AAalkAdn (for details see Figure 4).

All the constructs were verified by DNA sequencing.

### Survival and mutagenesis assays

To estimate the survival of tested strains, bacteria were grown in *E medium* to OD_600_ = 0.5-0.6, treated with MMS (20 or 50 mM) or MNNG (2 or 5 µg/ml) for indicated times (5 to 60 min), centrifuged, washed, suspended in the same volume of fresh medium, diluted, and plated on LB plates. After one day of incubation, the colonies of viable cells were counted and the percent of survivors calculated.

The sensitivity of bacteria to MMS and MNNG was also examined by employing the drop test. *P. putida* strains were grown overnight or until mid-exponential phase in liquid LB medium. Bacterial cultures were then serially diluted into M9 medium, and diluted cultures (1µl) were spotted onto agar plates containing LB or LB supplemented with MMS (0.5 mM) or MNNG (0.74 µg/ml). Images were taken after two days of incubation of bacteria at 30°C.

To test for mutagenicity, MMS (20 mM)- or MNNG (2 µg/ml for *P. putida* and 5 µg/ml for *E. coli*)-treated bacteria and non-treated controls were diluted 1:10 in *E medium*, grown overnight to express the mutations, and plated on LB plates for viable cells and on LB plates containing rifampicin for Rif^R^ mutants after one day of incubation at 30°C. Following the counts, the frequencies of Rif^R^ mutants (number of Rif^R^ mutants per 10^8^ cells) were calculated.

For complementation assays, *E. coli* AB1157 alkB‾ strain DM12 harboring the pVB1x plasmid expressing *P. putida* or *E. coli alkB* (“empty” pVB1x plasmid served as a control) were treated for 15 min with 20 mM MMS, centrifuged, washed with C salts, resuspended and then diluted 1:10 in *E medium*, grown overnight, and plated on LB plates for viable cells (one day of incubation) and on E-Arg plates for Arg^+^ revertants (two days of incubation). Following the counts, the frequencies of Arg^+^ reversions (number of Arg^+^ revertants per 10^8^ cells) were calculated.

### Phage survival assay

For the phage survival test, M13 (ssDNA) and MS2 (ssRNA) phages were used. The *E. coli* alkB‾ F^+^ strain bearing the pVB1x “empty” plasmid as a control or the plasmid expressing EcAlkB or PpAlkB was grown to stationary phase in E-Pro medium supplemented with kanamycin (50 µg/ml) and carbenicillin (100 µg/ml), and diluted 30-fold in fresh medium. At OD_600_ = 0.2 and 0.8, 2 mM toluic acid and 5 mM MgCl_2_ were added, respectively. Then, 150 µl of bacteria was mixed with 100 µl of phage preparation, incubated for 10 min at 37°C, mixed with 3 ml LCA prewarmed to 45°C and poured onto LB plates. Plaques were counted after 5-8 h (MS 2) or 18-24 h (M13) of incubation at 37°C and PFUs (Plaque Forming Units per ml) were calculated. To modify the phages, M13 or MS2 suspensions in C salts were treated with 15 mM MMS or 20 mM CAA for 30 min at 30°C.

### Expression, purification, and *in vitro* activity assays of PpAlkB protein

#### PpAlkB expression and purification

The pET28(a):PpalkB construct was introduced into *E. coli* BL21 pLysS strain. The bacteria were grown in 1 L of LB supplemented with kanamycin (100 µg/ml) and chloramphenicol (20 µg/ml) at 37°C. At the OD_600_ of ~ 0.6, the culture was cooled to 16°C and expression of PpAlkB was induced with 0.1 mM IPTG. After overnight at 16°C, bacteria were spun down (15 min, 6000 rpm, 4°C) and sonicated in 45 ml of Lysis Buffer (50 mM Tris-HCl pH 8.0, 500 mM NaCl, 20 mM imidazol, 100 µM PMSF, 1 mM DTT, and 0.1% Triton X-100). The lysate was centrifuged (45 min, 12,000 rpm, 4°C) and the supernatant incubated with 400 µl of 50% His-Select-Nickel Affinity Gel (Sigma-Aldrich). The resin was washed with Lysis Buffer and the protein eluted with Elution Buffer (Lysis Buffer supplemented with 300 mM imidazol). Protein was dialyzed against Dialysis Buffer (50 mM Tris-HCl pH 8, 300 mM NaCl, 100 µM PMSF, 1 mM DTT, and 25% glycerol).

#### Preparation of AlkB substrates

Substrates were prepared as in ref [20,22]. . Briefly, pentadeoxynucleotides, TTXTT, where X is cytosine (C) or adenine (A), were reacted with chloroacetaldehyde (CAA), acrolein (ACR) or methyl methanesulfonate (MMS) obtaining derivatives containing modified cytosines: HEC, εC, 3meC, and HPC or adenine: εA. After HPLC purification, the modified pentamers were used as AlkB substrates.

#### AlkB assay

The 20 µL of reaction mixtures contained 50 mM appropriate buffer, 1 mM DTT, different concentrations of Fe(NH_4_)_2_(SO_4_)_2_, 50 µM 2-oxoglutarate, and 0.5 nmol of the substrate. Reactions were started by the addition of purified Pp or EcAlkB protein or the studied component, allowed to proceed at various temperatures (as indicated in the text and figure legends), stopped by the addition of 230 µl ice-cold water, frozen at -20°C to deactivate the enzyme, and then analyzed by HPLC. The Fe(II) ion concentrations were adjusted [22] according to concentrations of H^+^ and Fe(II) cations optimal for the repair of the studied adducts by the EcAlkB protein.

### Promoter induction assay

To study the activity of *P. putida ada, alkB* and *alkA* as well as *E. coli ada* promoters, the pPROBE-NT:*ada*p, pPROBE-NT:*alkB*p pPROBE-NT:*alkA*p and pPROBE-NT:*Ecada*p constructs were introduced into *P. putida* KT2440 wild-type cells by electrotransformation. The pPROBE-NT (promoterless) and pPROBE-KT:*kan*p (kanamycin promoter) were used as negative and positive controls, respectively. Two isolates were tested for promoter induction. The overnight LB cultures of *P. putida* strains harboring selected plasmids were used to inoculate *E medium* and were grown to OD_600_ 0.5-0.6. At this point, 20 mM MMS or 2 or 5 µg/ml MNNG was added. After 15 min of incubation, bacteria were spun down (5000 rpm, 4 min), suspended in fresh *E medium* (supplemented with kanamycin if needed) and cultured at 30°C with shaking. The fluorescence level of synthesized GFP was measured in 100 µl of bacterial culture placed in 96-well plates in triplicate. Fluorescence was induced at 485/20 nm and detected at 528/20 nm (sensitivity set at 50), whereas cell density was measured by absorbance at 600 nm using Synergy HTI Microplate Reader (Biotech). Additionally, the above constructs were tested analogously in *E. coli* AB1157 strain. For *P. putida* ada‾ strain, the pPROBE-AT backbone was used instead of pPROBE-NT.

To normalize the fluorescence, the absolute fluorescence (F_a_) was divided by absorbance value (OD_600_), giving Relative Fluorescence Units: RFU = F_a_/OD_600_. To eliminate the impact of MMS and MNNG (methylation reaction) on GFP fluorescence level, the fluorescence of recombinant eGFP protein (Clontech) was measured in the presence of 50 mM MMS or 5 µg/ml MNNG, with no change in fluorescence levels.

### Sequence alignments and analysis of the *P. putida* Ada regulon

#### *Ada regulon* organization

The genome sequences of *E. coli* K-12 DH10B (NC_010473), K-12 MG1655 (NC_000913), BW2952 (NC_012759), B REL606 (NC_012967), CFT073 (NC_004431), HS (NC_009800), O157:H7 EC4115 (NC_011353), and SE11 (NC_011415) and *P. putida* KT2440 (NC_002947) [64], F1 (NC_009512), GB-1 (NC_010322), S16 (NC_015733), BIRD-1 (NC_017530), ND6 (NC_017986), DOT-T1E (NC_018220), HB3267 (NC_019905), H8234 (NC_021491), NBRC 14164 (NC_021505), and W619 (NC_010501) were retrieved from NCBI database. The particular genes were identified by BLAST [65] as well as with Conserved Domain databases [66] at NCBI (AlkB: COG3145; AlkA: COG0122; AlkA-N: cl05528; Tag: COG2818; Aag: cd00540; AidB: cd01154). All manipulations were done and pictures prepared with Geneious 6.1.5 (Biomatters, http://www.geneious.com).

#### *P. putida* and *E. coli* alkyl damage glycosylases and acyl-CoA dehydrogenases proteins clustering 

To analyze the relationship between *P. putida* AlkA2 and Tag2 proteins, the CLANS [67] protein clustering of *P. putida* and *E. coli* glycosylases coping with alkyl damages was performed with Aag Representatives (family cd00540, NCBI) using the sequences in File S1.

To identify putative *aidB* genes in *P. putida* KT2440 genome, the acyl-CoA dehydrogenases of *P. putida* and *E. coli* species were retrieved from the protein family cd01154 (NCBI). This family consists of mean number of 15 and 4 AidB sequences for each *P. putida* and *E. coli* species, respectively. These sequences were grouped into fasta file (File S2) and CLANS [67] analysis was performed. Additionally, the search in MicrobesOnline database (http://www.microbesonline.org [64]) was undertaken using as a query the *E. coli* K-12 DH10B *aidB* genomic context, giving as a result the *P. putida* KT2440 PP_0158 locus – putative *gcdH* dehydrogenase.

#### Sequence alignment of AlkA and Ada proteins 

*P. putida* KT2440 (PP_0705; Gene ID: 1044536)*, Deinococcus radiodurans* R1 (DR_2584; Gene ID: 1797538) and *E. coli* K-12 DH10B (ECDH10B_2218; Gene ID: 6059665) AlkAs were aligned with *P. putida* GB-1 (PputGB1_2545; Gene ID: 5870334) AlkA2 using ClustalW [68] with the following parameters: Cost matrix – ID, Gap open cost -15, Gap extend cost -0.5. Domain searches were performed with InterProScan and Pfam domains were visualized. The ClustalW alignment and InterProScan search tools are implemented in Geneious Pro 6.1.5, and were used as such. Also the *P. putida* and *D. radiodurans alkA* with genetic context 500 bp upstream and downstream were checked for the presence of longer ORFs and the lengths of current ORFs were confirmed.

The *P. putida* (PP_0706; Gene ID: 1044537) and *E. coli* K-12 DH10B (ECDH10B_2370; Gene ID: 6059404) Ada proteins were aligned with ClustalW [68] with the following parameters: Cost matrix – ID, Gap open cost -10, Gap extend cost -0.1.

#### Analysis of promoter regions of ada, alkA, alkB, and aidB genes 

The promoter sequences (-100, -1) of *ada*, *alkA*, *alkB* and *aidB*, genes of above mentioned 8 *E. coli* and 5 *P. putida* strains were retrieved from genomic sequences and grouped into one file (File S3). Additionally, the *P. putida* GB-1 (PputGB1_2545; Gene ID: 5870334) and S16 (PPS_2874; Gene ID: 10938506) *alkA2* promoter sequences were added. Although *alkA2* genes are annotated as alcohol dehydrogenases, the protein sequence Conserved Domain search [66] shows AlkA domain architecture (COG0122), additionally, the BLAST search [65] against *E. coli* K-12 DH10B proteome reveals the identity of *P. putida* GB-1 and S16 AlkA2 proteins with the *E. coli* AlkA at the level of 36 and 37%, respectively.

The motif files (Files S4 and S5) were generated with MEME tool [69] with *E. coli* K-12 DH10B *ada*, *alkA*, and *aidB* promoter consensus sequences used as inputs. These were subsequently used as inputs for the FIMO tool [70], allowing the search in File S3 database with *P*-value better than 0.01. Thus, the obtained output files (Files S6 and S7) were fused, the same sequences removed, and formatted for fasta file format (File S8). Subsequently, this file was used to generate phylogenetic tree with Geneious Tree Builder with the following parameters: Cost Matrix – Identity (1.0/0.0), Gap open penalty -12, Gap extension penalty -3, Alignment type – Global alignment, Genetic Distance Model – Tamura-Nei, Tree build Method – UPGMA. All the sequence alignments and tree manipulations were performed in Geneious 6.1.5 (Biomatters, http://www.geneious.com).

The borders of the consensus sequences in Ada regulon promoters responsible for Ada protein specific interaction were established according to ref [30]. . Additionally, the positions of -35 and -10 boxes for RNA polymerase in *E. coli ada*, *alkA*, and *aidB* promoters were retrieved from RegulonDB (http://regulondb.ccg.unam.mx) or predicted for *P. putida alkA*, *aidB*, and *alkB* promoters according to SoftBerry BPROM prediction tool (http://linux1.softberry.com).

### Statistical analysis

Survival and mutagenesis experiments were repeated 4-10 times, each in duplicate, and standard deviation (± SD) was calculated. Statistically important differences were tested on the basis of Student t-test (*P* < 0.05, two-sided, implication of different variances). Counts were computed and graphs were constructed using SigmaPlot (Systat Software, San Jose, CA). Figures 1, 2, 3 and 4 were prepared with GIMP 2.8 image manipulation software.

## Supporting Information

File S1 **The fasta file of *P. putida* and *E. coli* alkyl damage glycosylases with Aag Representatives (family cd00540, NCBI).**
(FASTA)

File S2 **The fasta file of *P. putida* and *E. coli* acyl-CoA dehydrogenase sequences retrieved from AidB family cd01154 (NCBI).**
(FASTA)

File S3 **The fasta file of *P. putida* KT2440, F1, S16, GB-1, and W619 and *E. coli* K-12 DH10B, K-12 MG1655, B REL606, BW2952, CFT073, HS, O175:H7 EC4115, and SE11 strains of *ada*, *alkA*, *aidB*, and *alkB* promoter sequences (from -100 to -1 relative to ATG start codons).**
(FASTA)

File S4 **The MEME output file for *E. coli* K-12 DH10B *ada*, *alkA*, and *aidB* promoter consensus sequences used as input.**
(TXT)

File S5 **The MEME output file for *P. putida* KT2440, F1, and W619 *alkA* and KT2440, F1, GB-1, and GB-1 *aidB* promoter consensus sequences used as input.**
(TXT)

File S6 **The FIMO output search results with File S4 as motif file and File S3 as database input file.**
(TXT)

File S7 **The FIMO output search results with File S5 as motif file and File S3 as database input file.**
(TXT)

File S8 **The fasta file of FIMO outputs (Files S6 and S7) with the sequences redundant at 100% removed.**
(FASTA)

Table S1 **Primers used in the study for the preparation of *P. putida* deletion strains.**
(DOCX)

Table S2 **Primers used in the study for PpalkB (Pputup, Pputdn) and promoter sequences cloning (PpalkAH1, PpalkAdE; PpalkBH1, PpalkBdE; PpadauH1, PpadadnE; Ecadaru, Ecadard) or used for PpalkA promoter deletion/mutation (AalkAup, AalkAdn; BalkAup, BalkAdn; ABalkAup, ABalkAdn; AmalkAup, AmalkAdn; AAalkAup, AAalkAdn).**
(DOCX)
