# Supplementary material for: Pseudomonas putida AlkA and AlkB Proteins Comprise Different Defense Systems for the Repair of Alkylation Damage to DNA – In Vivo, In Vitro, and In Silico Studies
Source: PLoS One. 2013 Oct 2;8(10):e76198. doi: 10.1371/journal.pone.0076198 (PMC3788762; doi:10.1371/journal.pone.0076198)
Supplement: Table S1 — Primers used in the study for the preparation of P. putida deletion strains. (DOCX) [file pone.0076198.s009.docx]

**Table S1.** Primers used in the study for the preparation of *P. putida* deletion strains.

________________________________________________________________________________

Primer name Primer sequence Complementary region

________________________________________________________________________________

ppAlkBFwSacuus 5´-ATATGAGCTCAGAGCTACACCAA- Complementary to the

-CCAACTCTA-3´ sequence -350 to -328 upstream of the ATG initiator codon of *P. putida* *alkB* gene. The restriction site of SacI is underlined.

ppAlkBRevAccuus 5´-ATATGGTACCCGAGCTTGGCATTG- Complementary to the

-GCATTCCA-3´ sequence +275 to +254 downstream of the TGA stop codon of *P. putida* *alkB* gene. The restriction site of Acc65I is underlined.

ppAlkBlookusuus 5´-ACGAGCTGTACTTCGAACAGAA-3´ Complementary to the

sequence -486 to -464 upstream of the ATG initiator codon of *P. putida* *alkB* gene.

GmY 5′-CGAATTGACATAAGCCTGTTC-3′ Complementary to the

sequence -240 to -220 upstream of the ATG initiator codon of Gm^r^ gene.

GmA 5′-CGGCTTGAACGAATTGTTAG-3′) Complementary to the

sequence +528 to +509 downstream of the ATG initiator codon of Gm^r^ gene.

ppAdaFwSac 5´-ATATGAGCTCCGTACAGGCAACAAC- Complementary to the

-ATATCCG-3´ sequence -32 to -11 upstream of the ATG initiator codon of *P. putida ada* gene. The restriction site of SacI is underlined.

ppAdaRevXba 5´-ATATTCTAGAGCGGTCAGGAGAGTG- Complementary to the

-CCGTTT-3´ sequence -14 to +4 of the TGA stop codon were used of *P. putida* *ada* gene. The restriction site of XbaI is underlined.

ppadalookus 5´-GTACCGTGGCGGCCTGGTAC-3´ Complementary to the

sequence -82 to -63 upstream of the ATG initiator codon of *P. putida ada* gene.

KmSac 5´-CAGGAGCTCGTTCGATTTATTCAACA- Complementary to the

-AAGCC-3´ omega element next to Km^r^ gene. The restriction site of SacI is underlined.

KmH 5´-CTGCCAGTGTTACAA-3´ Complementary to the

sequence of Km^r^ gene region in positions of 31 to 17 from the stop codon TAG oriented towards the Km^r^ gene.

ppAlkA1SacIFw 5´-ATATGAGCTCAGCGCAACCGGTAGCAG-3´ Complementary to the

sequence -673 to -656 upstream of the ATG initiator codon of *P. putida* *alkA* gene. The restriction site of SacI is underlined.

ppAlkA1Rev 5´-TGTACGGGCGATGCGAAAATGCCATTG- Complementary to the

-CTCCGCGC-3´ sequence -87 to -103 upstream of the ATG initiator codon of *P. putida* *alkA* gene. The reverse complement sequence of primer ppAlkA2F is underlined.

ppAlkA2Fw 5´-TTTCGCATCGCCCGTACA-3´ Complementary to the

sequence +4 to +21 downstream of the TGA stop codon of *P. putida alkA* gene.

ppAlkA2XbaIRev 5´-ATATTCTAGAAGTATTGCACCCAG- Complementary to the

-CGAG-3´ sequence +645 to +628 downstream of the TGA stop codon of *P. putida alkA* gene. The restriction site of XbaI is underlined.

ppAlkAKI 5´-AGTCCAGTGATGTGTGCG-3´ Complementary to the

sequence – 157 to -140 upstream of the ATG initiator codon of *P. putida alkA* gene.

ppAlkAKII 5´-ATACCGCCAGCAACTGCT-3´ Complementary to the

sequence 263 to 245 downstream of the ATG initiator codon of *P. putida alkA* gene.

pSW-F 5´-GGACGCTTCGCTGAAAACTA-3´ Diagnose presence/loss pSW

plasmid [[1](#_ENREF_1)].

pSW-R 5´-AACGTCGTGACTGGGAAAAC-3´ Diagnose presence/loss pSW

plasmid [[1](#_ENREF_1)].

________________________________________________________________________________

1. Martinez-Garcia E, de Lorenzo V (2011) Engineering multiple genomic deletions in Gram-negative bacteria: analysis of the multi-resistant antibiotic profile of *Pseudomonas putida* KT2440. Environ Microbiol 13: 2702-2716.
