# Supplementary material for: Pseudomonas putida AlkA and AlkB Proteins Comprise Different Defense Systems for the Repair of Alkylation Damage to DNA – In Vivo, In Vitro, and In Silico Studies
Source: PLoS One. 2013 Oct 2;8(10):e76198. doi: 10.1371/journal.pone.0076198 (PMC3788762; doi:10.1371/journal.pone.0076198)
Supplement: Table S2 — Primers used in the study for PpalkB (Pputup, Pputdn) and promoter sequences cloning (PpalkAH1, PpalkAdE; PpalkBH1, PpalkBdE; PpadauH1, PpadadnE; Ecadaru, Ecadard) or used for PpalkA promoter deletion/mutation (AalkAup, AalkAdn; BalkAup, BalkAdn; ABalkAup, ABalkAdn; AmalkAup, AmalkAdn; AAalkAup, AAalkAdn). (DOCX) [file pone.0076198.s010.docx]

**Table S2.** Primers used in the study for PpalkB (Pputup, Pputdn) and promoter sequences (PpalkAH1, PpalkAdE; PpalkBH1, PpalkBdE; PpadauH1, PpadadnE; Ecadaru, Ecadard) cloning or used for PpalkA promoter deletion/mutation (AalkAup, AalkAdn; BalkAup, BalkAdn; ABalkAup, ABalkAdn; AmalkAup, AmalkAdn; AAalkAup, AAalkAdn).

| Primer name | Sequence (5’→3’) | Restriction site |
| --- | --- | --- |
| Pputup  Pputdn | CTGAATCATATGATCCAGTCCGACCTCGAC  CAATGCCTCGAGTCAGGCACCCGCCTTGCGCAA | NdeI  XhoI |
| PpalkBH1  PpalkBdE | AGTTCAAGCTTCATCCCGCCCTCTGCCATCGTTCGA  AGTTCGAATTCGTGGCTGCACATCTTCGAGCGGTT | HindIII  EcoRI |
| PpadauH1  PpadadnE | AGTTCAAGCTTGCGCCTGGCGGCCGTTTCGTACC  AGTTCGAATTCCTTTGTTTCCCGGATATGTTGTTG | HindIII  EcoRI |
| PpalkAH1  PpalkAdE | AGTTCAAGCTTCGGAGCAATGGCATGCCAAAGAT  AGTTCGAATTCGGCACCTCCATCAAGTCGATTGC | HindIII  EcoRI |
| Ecadaru  Ecadard | AGGAATTGGGGATCGGAAGCTTAGCTTCCTTGTCAGCGAAAAAAATTAAAG  ATCTTTAGTTAGTTAGGGAATTCAATCAGCTCCCTGGTTAAGGATAG | HindIII  EcoRI |
| AalkAup  AalkAdn | GCAATGGCATGCCAAAGAGCTGCGCAAGAACCG  CGGTTCTTGCGCAGCTCTTTGGCATGCCATTGC | - |
| BalkAup  BalkAdn | CAAAGATTAGCTGCGAACCGGCGTTTG  CAAACGCCGGTTCGCAGCTAATCTTTG | - |
| ABalkAup  ABalkAdn | GCAATGGCATGCCAAAGGAACCGGCGTTTGCCCG  CGGGCAAACGCCGGTTCCTTTGGCATGCCATTGC | - |
| AmalkAup  AmalkAdn | GCAATGGCATGCCAAAGGCCAGCTGCGCAAGAACCG  CGGTTCTTGCGCAGCTGGCCTTTGGCATGCCATTGC | - |
| AAalkAup  AAalkAdn | TCGGAGCAATGGCATGCCGCTGCGCAAGAACCGGC  GCCGGTTCTTGCGCAGCGGCATGCCATTGCTCCGA | - |
